# Population Structure of Hispanics in the United States: The Multi-Ethnic Study of Atherosclerosis

**DOI:** 10.1371/journal.pgen.1002640

**Published:** 2012-04-12

**Authors:** Ani Manichaikul, Walter Palmas, Carlos J. Rodriguez, Carmen A. Peralta, Jasmin Divers, Xiuqing Guo, Wei-Min Chen, Quenna Wong, Kayleen Williams, Kathleen F. Kerr, Kent D. Taylor, Michael Y. Tsai, Mark O. Goodarzi, Michèle M. Sale, Ana V. Diez-Roux, Stephen S. Rich, Jerome I. Rotter, Josyf C. Mychaleckyj

**Affiliations:** 1 Center for Public Health Genomics, University of Virginia, Charlottesville, Virginia, United States of America; 2 Department of Public Health Sciences, Division of Biostatistics and Epidemiology, University of Virginia, Charlottesville, Virginia, United States of America; 3 Department of Medicine, Columbia University, New York, New York, United States of America; 4 Department of Medicine and Department of Epidemiology, Wake Forest University School of Medicine, Winston-Salem, North Carolina, United States of America; 5 Department of Medicine, Division of Nephrology, University of California San Francisco, San Francisco, California, United States of America; 6 Division of General Internal Medicine, San Francisco VA Medical Center, San Francisco, California, United States of America; 7 Department of Public Health Sciences, Wake Forest University School of Medicine, Winston-Salem, North Carolina, United States of America; 8 Medical Genetics Institute, Cedars-Sinai Medical Center, Los Angeles, California, United States of America; 9 Department of Biostatistics, School of Public Health, University of Washington, Seattle, Washington, United States of America; 10 Department of Laboratory Medicine and Pathology, University of Minnesota, Minneapolis, Minnesota, United States of America; 11 Department of Medicine and Department of Biochemistry and Molecular Genetics, University of Virginia, Charlottesville, Virginia, United States of America; 12 Department of Epidemiology, Center for Social Epidemiology and Population Health, University of Michigan, Ann Arbor, Michigan, United States of America; Vanderbilt University School of Medicine, United States of America

## Abstract

Using ∼60,000 SNPs selected for minimal linkage disequilibrium, we perform population structure analysis of 1,374 unrelated Hispanic individuals from the Multi-Ethnic Study of Atherosclerosis (MESA), with self-identification corresponding to Central America (n = 93), Cuba (n = 50), the Dominican Republic (n = 203), Mexico (n = 708), Puerto Rico (n = 192), and South America (n = 111). By projection of principal components (PCs) of ancestry to samples from the HapMap phase III and the Human Genome Diversity Panel (HGDP), we show the first two PCs quantify the Caucasian, African, and Native American origins, while the third and fourth PCs bring out an axis that aligns with known South-to-North geographic location of HGDP Native American samples and further separates MESA Mexican versus Central/South American samples along the same axis. Using k-means clustering computed from the first four PCs, we define four subgroups of the MESA Hispanic cohort that show close agreement with self-identification, labeling the clusters as primarily Dominican/Cuban, Mexican, Central/South American, and Puerto Rican. To demonstrate our recommendations for genetic analysis in the MESA Hispanic cohort, we present pooled and stratified association analysis of triglycerides for selected SNPs in the *LPL* and *TRIB1* gene regions, previously reported in GWAS of triglycerides in Caucasians but as yet unconfirmed in Hispanic populations. We report statistically significant evidence for genetic association in both genes, and we further demonstrate the importance of considering population substructure and genetic heterogeneity in genetic association studies performed in the United States Hispanic population.

## Author Summary

Using genotype data from about 60,000 distinct genetic markers, we examined population structure in 1,374 unrelated Hispanic individuals from the Multi-Ethnic Study of Atherosclerosis (MESA), with self-identification corresponding to Central America (n = 93), Cuba (n = 50), the Dominican Republic (n = 203), Mexico (n = 708), Puerto Rico (n = 192), and South America (n = 111). By comparing genetic ancestry of MESA Hispanic participants to reference samples representing worldwide diversity, we show major differences in ancestry of MESA Hispanics reflecting their Caucasian, African, and Native American origins, with finer differences corresponding to North-South geographic origins that separate MESA Mexican versus Central/South American samples. Based on our analysis, we define four subgroups of the MESA Hispanic cohort that show close agreement with the following self-identified regions of origin: Dominican/Cuban, Mexican, Central/South American, and Puerto Rican. We examine association of triglycerides with selected genetic markers, and we further demonstrate the importance of considering differences in genetic ancestry (or factors associated with genetic ancestry) when performing genetic studies of the United States Hispanic population.

## Introduction

Although epidemiologic studies often regard Hispanics in the United States as a homogenous group, U.S. Hispanics have a complex population structure comprised of many overlapping subgroups, and also vary markedly in environmental and cultural factors linked to country of origin and history of immigration to the United States. A widely recognized distinction from genetic analysis has been between Hispanics carrying primarily Caucasian and African ancestry, versus those having predominantly Caucasian and Native American ancestry [1], [2], [3], with little admixture observed between individuals of predominantly African versus Native American ancestry. In the MESA Hispanic cohort, previous work using 199 ancestry informative markers (AIMs) to estimate proportions of ancestry in a subset of 705 individuals identified strong differences in proportions of European, Native American, and African ancestry by self-identified country/region of origin, with Mexican/Central Americans having the highest proportions of Native American ancestry, Puerto Ricans having the highest European ancestry, and Dominicans the highest African ancestry [3]. Recent studies have also documented diversity and population substructure within the Native American founder populations [4].

The Multi-Ethnic Study of Atherosclerosis (MESA) provides one of the largest and most thoroughly-characterized samples of Hispanic individuals to date. MESA has 1,374 unrelated Hispanic individuals and a total of 2,174 subjects of self-reported Hispanic ethnicity, including pedigrees. Most self-reported Hispanic participants also reported more detailed self-identification corresponding to Central America, Cuba, the Dominican Republic, Mexico, Puerto Rico or South American origin (Table S1). As MESA participants, each of these individuals was assessed for subclinical cardiovascular disease and risk factors that predict progression to clinically overt cardiovascular disease. In addition, genome-wide genotyping of >800,000 SNPs was performed for each of these individuals through the NHLBI SHARe program (MESA SHARe). These valuable phenotypic and genotypic data provide opportunities to perform Genome-Wide Association (GWA) studies for many cardiovascular phenotypes. Proper GWA analysis of the MESA Hispanic cohort requires a clear understanding of the population structure of Hispanics in the United States.

Using the recently available genome-wide genotype data, we perform population structure analysis of an unrelated subset of 1,374 individuals from the MESA Hispanic cohort. By Principal Component Analysis (PCA) [5], [6] and model-based cluster analysis [7], [8], we identify clear patterns of diversity across the MESA Hispanic cohort. We further draw on samples from the HapMap phase III [9] and Human Genome Diversity Panel (HGDP) [10], [11], representing worldwide genetic diversity including European, African, and Native American samples, to inform our population structure analysis. By combining dense genotype data from MESA SHARe with the available worldwide reference panels, we achieve greater resolution in examining intra-continental diversity, particularly among Native American ancestral populations.

We perform cluster analysis on the first four principal components (PCs) of ancestry to identify four distinct subgroups of the MESA Hispanic cohort. Based on participant self-identification, we find these subgroups represent primarily Central/South America, the Dominican Republic and Cuba, Mexico, and Puerto Rico. To demonstrate a principled approach to genetic association analysis taking into account genetic diversity in the MESA Hispanic cohort, we perform analysis of SNPs in the lipoprotein lipase (*LPL*) and tribbles homolog 1 (*TRIB1*) gene regions with triglycerides in the full MESA Hispanic cohort, as well is in stratified analyses to assess evidence for association within each of the four Hispanic subgroups. Our genetic analysis indicates pooled analysis provides the best power when there is only modest heterogeneity in genetic effects, while stratified analysis offers better resolution to detect genetic loci in which SNP effects are limited to or much stronger within a single subgroup of Hispanics.

## Results

### Principal component analysis

Principal components (PCs) of ancestry were computed for 1,374 unrelated individuals from the MESA Hispanic cohort using the program SMARTPCA, which is distributed with the software package EIGENSTRAT [5], [6]. The individuals included in the analysis represented six major countries/regions of origin: Central America, Cuba, the Dominican Republic, Mexico, Puerto Rico, and South America, with the exact counts detailed in Table S1. The principal component analysis was performed using 64,199 autosomal SNPs typed through MESA SHARe, with SNPs selected for minimal linkage disequilibrium (LD) among MESA Hispanics, and availability of genotypes in the HapMap phase III and HGDP reference panels.

**Figure 1:**
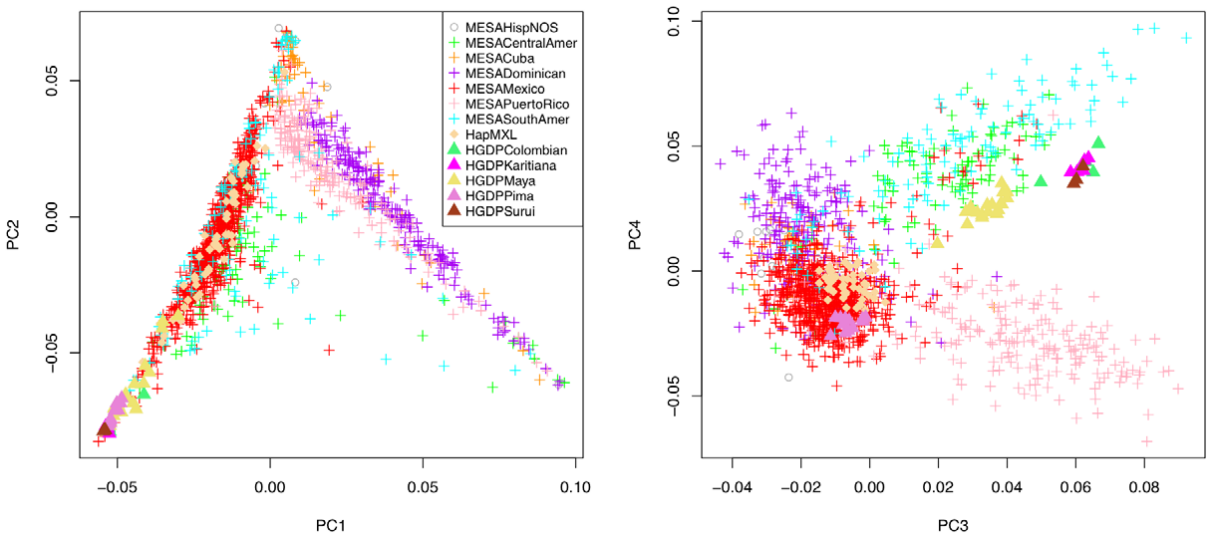
Principal component analysis of 1,374 unrelated individuals of self-reported Hispanic origin from the Multi-Ethnic Study of Atherosclerosis (MESA), displayed by country/region of origin, with projection to key reference populations. Individuals are labeled according to group inclusion: MESAHispNOS="MESA Hispanic, Other or Unspecified country/region of origin”, other labels are self-explanatory.

The resulting PCs were projected to HapMap phase III and HGDP samples, and the first four principal components of ancestry are displayed for an unrelated set of MESA Hispanic subjects and key reference populations in Figure 1. Among the many diverse populations in these reference panels, the HapMap phase III includes a sample of 30 unrelated individuals of Mexican ancestry from Los Angeles, California (MXL), while the HGDP includes 29 unrelated Native American individuals, further classified as either Colombian, Karitiana, Maya, Pima, or Surui. A geographic representation [10] of the sampling locations of the HGDP Native American individuals indicates they span Northern Mexico (Pima), Southern Mexico (Maya), the region of Colombia near the border with Brazil (Colombian), and Southwestern Brazil (Karitiana and Surui). These Native American samples provide a valuable resource to inform potential differences in Native American ancestry across the MESA Hispanic cohort. That said, there are notable gaps in coverage provided by the HGDP with, for example, no representation of Taino Arawaks, widely noted as a major source of Native American ancestry for present day Caribbean Hispanics [12]. Indeed, there is a practical limitation to obtaining genetic samples from Taino Arawaks (as well as other Native American founder populations) because few or no individuals survived past the period of European colonization.

The first two PCs of ancestry display strong population stratification across the Hispanic cohort. The three predominant sources of ancestry correspond to Caucasian, Native American and African founder populations, with the vast majority of MESA Hispanic individuals lying along two edges of a triangle, corresponding to two major clusters broadly representing individuals reporting Mexican versus Caribbean (Puerto Rican, Dominican or Cuban) origin. Projection of these principal components to all four MESA ethnic groups (Figure S1) as well as the worldwide diversity panels comprised of HapMap phase III and HGDP samples (Figure S2), we find the Mexican cluster predominantly represents admixture of Caucasian and Native American ancestry, while the Caribbean cluster reflects admixture of Caucasian and African ancestry. Although these two clusters are remarkably well separated from one another, evidence for Native American ancestry among Caribbean Hispanics is reflected in the plot of PC2 versus PC1. This evidence emerges forth when the PCs of Hispanics are viewed together with those of African Americans (Figures S1 and S2) who populate a more extreme (*i.e.* less admixed) position on the plot.

The plot of the third and fourth PCs reveals additional structure, separating Puerto Rican and Central/South American subjects into two distinct groups that are further separated from the rest of the MESA Hispanic cohort. Interestingly, population structure shown in the plot of PC4 versus PC3 is specific to MESA Hispanic and HGDP Native American samples, with little separation of other worldwide populations (Figures S1 and S2). A linear axis defined by PC3 and PC4 aligns with South-to-North geography of HGDP Native American subgroups (Colombian, Karitiana, Maya, Pima and Surui) with the South American Colombian, Karitiana and Surui at one end and the North American Pima at the other. The same axis corresponds closely with Mexican versus Central/South American origin, building on previous evidence that geographic and genetic distance show good correlation among Native Americans [13], and supporting the natural hypothesis that diverse Native American founder populations contributed to present day Hispanic populations in these regions. None of the available reference panels aligned with the Caribbean (Puerto Rican, Dominican or Cuban) samples along the third and fourth principal components of ancestry, a reasonable result given none of the known Native American populations of the Caribbean region, such as Taino Arawaks [12], were included in the available reference panels [10]. These data suggest Native American founders contributing to present day Caribbean populations are genetically distinguishable from those in Mexico and Central/South American.

We did not identify any clear patterns of population substructure in the MESA Hispanic cohort in plots of the higher order PCs (Figures S1 and S2). We further examined the proportion of variance explained by the strongest PCs of ancestry. The first four PCs of ancestry explained 1.90%. 0.85%, 0.141% and 0.125% of variance, respectively, compared to 0.093%–0.109% of variance explained by each of the remaining PCs corresponding to the largest 100 eigenvalues from the PCA. Based on this combination of evidence from the scatter plots and eigenvalues from PCA, we determined it was sufficient to focus subsequent genetic analyses on the first four PCs of ancestry.

### Model-based structure analysis

**Figure 2:**
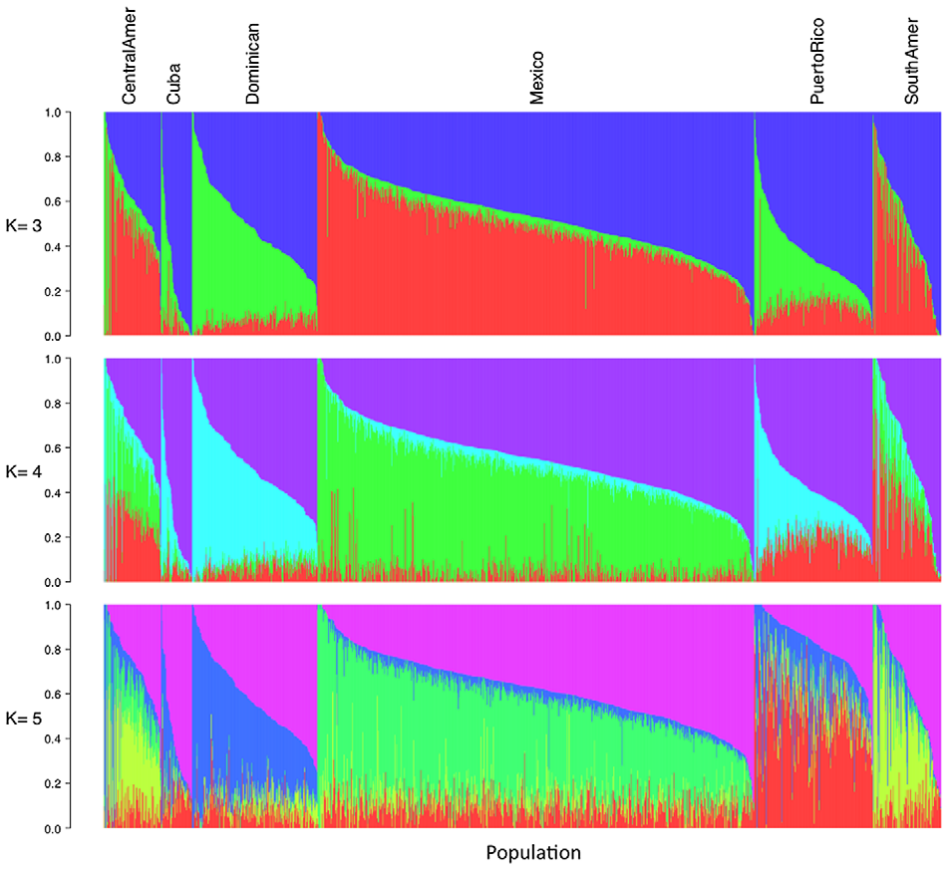
Illustration of model-based clustering results from ADMIXTURE, based on 1,374 unrelated individuals of self-reported Hispanic origin from the Multi-Ethnic Study of Atherosclerosis (MESA), shown for K = 3, 4, and 5. Results are displayed only for individuals from MESA whose self-reported country/region of origin was reported unambiguously as Central America, Cuba, Dominican Republic, Mexico, Puerto Rico, or South America.

Using the same set of 1,374 unrelated individuals from the MESA Hispanic cohort and the same 64,199 autosomal SNPs as used for PCA, we performed model-based cluster analysis using the software ADMIXTURE [7]. We performed analysis for K = 2 to K = 7 distinct ancestral populations. Keeping in mind that the model-based cluster analysis does not make use of the self-identified country/region of origin information available through MESA, we see remarkable structure in the results plotted by region (Figure 2, Figure S3). For K = 3, the putative Caucasian ancestral population accounts for a considerable proportion of ancestry across all countries/region of origin, ranging from 37% in Central Americans to 73% among Cubans, while the putative African ancestral population accounts for as much as 43% of ancestry overall in Dominicans, and as little as 4% of overall ancestry among Mexicans. For K = 3, a third group corresponds to the Native American ancestry population, accounting for only 6% of ancestry overall in Cubans and Dominicans, and as much as 45 and 48% in Central Americans and Mexicans, respectively (Table 1). We also note considerable diversity within each country/region of origin with, for example, 34% of Cubans having greater than 90% Caucasian ancestry, while another 15% of Cubans have less than 50% Caucasian ancestry.

**Table 1:** Proportion of ancestry estimates averaged within each Hispanic country/region of origin, from model-based clustering analysis of 1,374 unrelated MESA individuals in ADMIXTURE with K = 3, 4, and 5.

		Self-reported Hispanic country/region of origin					
		CentralAmer	Cuba	Dominican	Mexico	PuertoRico	SouthAmer
**K = 3**	Caucasian	0.37	0.73	0.50	0.47	0.62	0.50
	African	0.18	0.21	0.43	0.04	0.25	0.11
	Native American	0.45	0.06	0.06	0.48	0.13	0.40
**K = 4**	Caucasian	0.31	0.70	0.47	0.45	0.56	0.42
	African	0.17	0.21	0.43	0.04	0.24	0.10
	Native American 1	0.26	0.04	0.04	0.46	0.02	0.16
	Native American 2	0.26	0.05	0.06	0.04	0.17	0.32
**K = 5**	Caucasian	0.26	0.60	0.42	0.38	0.18	0.36
	African	0.17	0.20	0.43	0.04	0.22	0.10
	Native American 1	0.17	0.03	0.02	0.43	0.03	0.06
	Native American 2	0.33	0.04	0.06	0.06	0.08	0.39
	Native American 3	0.07	0.13	0.07	0.09	0.49	0.08

Inferred ancestral populations from ADMIXTURE analysis are labeled based on putative assignments (*e.g.* Caucasian, African or Native American), as interpreted by the authors.

For K = 4 and K = 5, the first two groups correspond to Caucasian and African ancestral populations as seen for K = 3, while additional ancestral populations appear to account for regional differences in Native American ancestry (Table 1, Figure 2). Comparing results from K = 3 and K = 4, we see remarkable agreement in the relative proportions of Caucasian, African and Native American ancestry across all Hispanic countries/regions of origin. However, K = 4 shows a very clear separation in assignment of Native American ancestry to distinct groups for individuals of self-identified Mexican versus Puerto Rican origin, with Central/South Americans demonstrating a mixture of these two Native American ancestral populations. Results from K = 5 suggest further separation in the Native American ancestral populations, with one group represented predominantly among Mexicans, one group predominantly among Puerto Ricans, and a third group represented primarily in Central/South Americans. Due to the relatively lower proportion of Native American ancestry among individuals of Cuban and Dominican origin, it is difficult to comment definitively on their sources of Native American ancestry.

### Cluster analysis to identify Hispanic subgroups

We performed k-means clustering using the first four principal components of ancestry, to define four major groups within the Hispanic cohort. The resulting clusters of ancestry showed notably good agreement with self-identified country/region of origin, and were accordingly identified with Central/South America (abbreviated “CSAmer”), the Dominican Republic and Cuba, Mexico, and Puerto Rico (Table 2).

**Table 2:** Descriptive summaries of groups obtained by k-means cluster analysis of the first four principal components of ancestry for individuals of self-identified Hispanic origin from the Multi-Ethnic Study of Atherosclerosis (MESA).

		Classification (based on k-means clustering)			
		CSAmer	Dominican/Cuba	Mexico	Puerto Rico
**Sex**	**(% Female)**	55.2	57.0	48.8	53.1
**Age (in years)**	**Median**	61	61	62	58
	**(IQR)**	(52–68)	(52–69.75)	(54–69)	(52–66.5)
**Self-reported Hispanic country/region of origin (N)**	**Central America**	77	14	2	0
	**Cuba**	0	49	0	1
	**Dominican Republic**	0	199	0	4
	**Mexico**	22	27	658	1
	**Puerto Rico**	1	18	0	173
	**South America**	81	30	0	0
	**Other/Not specified**	0	13	4	0
**Total**		181	350	664	179

Results are shown an unrelated subset of 1,374 unrelated individuals from the MESA Hispanic cohort. Groups are labeled (“CSAmer”, “Dominican/Cuba”, “Mexico” and “Puerto Rico”) based on overall representation of self-identified country/region of origin within each cluster.

Each of the clusters was labeled as such because it carried the vast majority of individuals self-identifying with the corresponding region, *i.e.* the Mexican cluster contained 658 of 708 unrelated individuals with Mexico as their self-identified country of origin. In most cases, it was also true that a given cluster carried very few individuals self-identifying with a different country/region of origin, with the Dominican/Cuban cluster being the one notable exception. The Dominican/Cuban cluster is labeled as such because it contains 199 of 203 self-identified Dominican individuals and 49 out of 50 self-identified Cuban individuals from the unrelated subset of individuals reported in Table 2. However, this cluster also includes fourteen to thirty unrelated individuals self-identifying with each of the following: Central America, Mexico, Puerto Rico, and South America. This result reflects the fact that the Dominican/Cuban cluster tends to capture individuals carrying relatively little Native American ancestry, with varying proportions of Caucasian and African ancestry. While this genetic profile is characteristic of individuals self-identifying as Dominican or Cuban in the MESA Hispanic cohort, such individuals are also found throughout Latin America.

### Genetic association of triglycerides for candidate gene regions

Multiple studies have reported association between SNPs in the lipoprotein lipase (*LPL*) and tribbles homolog 1 (*TRIB1*) gene regions with triglyceride levels in GWAS of Caucasians [14], [15], [16], [17], yet it remains unclear whether the same gene regions show association in Hispanics [18]. A recent paper probed association in samples of Mexican individuals for SNPs reported in these gene regions in GWAS of Caucasians, identifying suggestive, but not statistically significant evidence of association [18]. Here, we perform a more comprehensive study looking at an expanded set of SNPs across the more diverse set of individuals included in the MESA Hispanic cohort.

### Genetic association analysis of SNPs in the LPL gene region

We selected SNPs rs10096633 and rs12678919 reported in previous studies [14], [15], [16], [17], [18], and examined association between 33 SNPs in the MESA Hispanic cohort (8 genotyped and 25 imputed) that exhibited strong linkage disequilibrium (LD) with the *LPL* index SNPs in Caucasians. To assess association, we performed pooled analysis of MESA Hispanics (N = 1779), as well as stratified analysis within the PCA-based clusters corresponding to Central and South America (N = 204), the Dominican Republic (N = 472), Mexico (N = 913) and Puerto Rico (N = 181).

**Figure 3:**
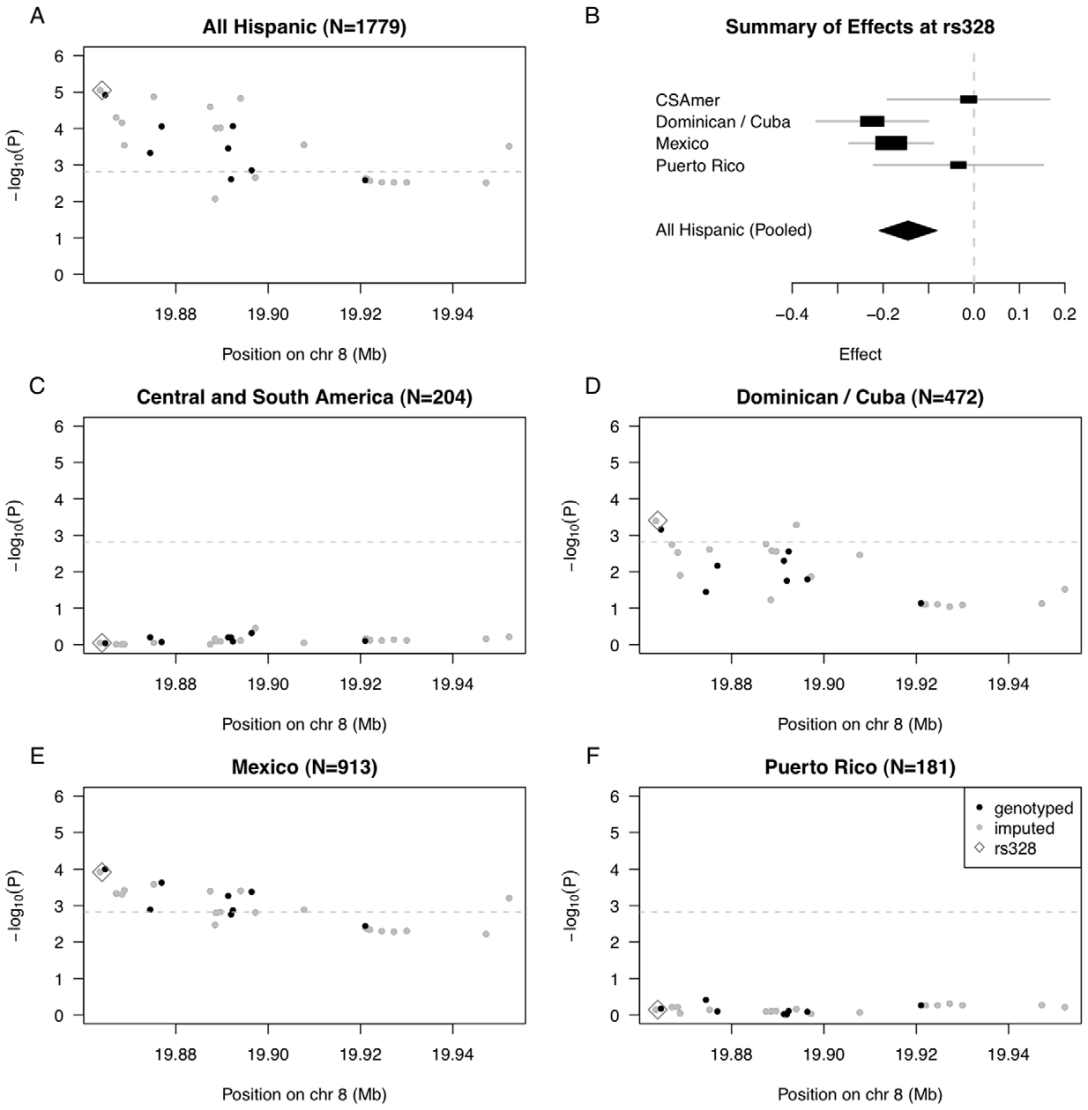
Summary of regional association for SNPs in the *LPL* gene region with triglycerides (modeled on a log scale). (A) Strength of association versus SNP position on chromosome 8 based on pooled analysis of MESA Hispanic individuals; (B) Forest plot of effects (with 95% CIs) reported in subsets of the MESA Hispanic cohort, using subgroups obtained from PCA-based cluster-analysis; and Strength of association versus SNP position on chromosome 8 based on stratified analysis of inferred clusters corresponding to (C) Central/South America, (D) the Dominican Republic and Cuba, (E) Mexico, and (F) Puerto Rico. In plots (A) and (C–F), genotyped SNPs are indicated as solid black dots, imputed SNPs as solid gray dots, the imputed SNP rs328 as an open gray diamond, and horizontal dashed gray lines indicate a conservative Bonferroni-threshold for statistical significance based on multiple testing of 33 SNPs.

In pooled analysis of the selected 33 *LPL* SNPs in MESA Hispanics, we saw statistically significant association of 18 SNPs with triglyceride outcomes (even after conservative Bonferroni correction for multiple testing using the cutoff 0.05/33 = 0.0015), with the strongest association observed for rs325, *P* = 8.86E-6, and rs328 (Ser474Stop), *P* = 8.88E-6 (Figure 3A, Table S2). Given the ancestral variability across Hispanic subgroups included in the pooled analysis, we further examined estimated effects of the functional SNP rs328 within each of our four PCA-based subgroups (Figure 3B). In stratified analysis, the Dominican/Cuban and Mexican subgroups had comparable estimated effects of −0.224 (SE = 0.063, coded allele freq. 0.073) and −0.182 (SE = 0.047, coded allele freq. 0.069) on log triglycerides (log mg/dL) per copy of the coded G allele, respectively. These estimated effect sizes are comparable to the value of −0.123 (SE = 0.025) previously reported as the estimate effect for the minor allele of the most strongly associated *LPL* region SNP rs10096633 in a GWAS of Caucasians [16]. In contrast, the estimated effects for Central/South American and Puerto Rican subgroups were closer to zero, with values −0.012 (SE = 0.091, coded allele freq. 0.077) and −0.034 (SE = 0.095, coded allele freq. 0.091), respectively.

To quantify evidence for heterogeneity in genetic effects of rs328 observed across the four Hispanic subgroups, we performed a test of genetic heterogeneity using the meta-analysis software METAL [20]. We do not find statistically significant evidence of heterogeneity (*P* = 0.13, heterogeneity I^2^ = 11.4), perhaps reflecting the fact that rs328 is a nonsense mutation, and is quite possibly a causal variant underlying the observed association. Still, we keep in mind the test of heterogeneity may be somewhat underpowered given the Central/South American and Puerto Rican subgroups have only ∼200 individuals each.

We went on to examine strength of association with each of the selected 33 SNPs in the *LPL* region, in stratified analyses of each of the four Hispanic subgroups (Figure 3C–3F; Tables S3, S4, S5, S6). We found statistically significant evidence of association for 17 SNPs in analysis of the Mexican subgroup, and for 4 SNPs in analysis of the Dominican/Cuban subgroup, but nothing close to suggestive for the Central/South American and Puerto Rican subgroups (Tables S3 and S6). Our genetic analysis of the *LPL* gene region demonstrates that when there is only modest genetic heterogeneity across the Hispanic cohort for a given locus, pooled analysis will tend to provide a stronger signal than any subgroup alone.

We performed genetic association analyses stratified by self-reported country/region of origin to provide a direct comparison with our stratified analyses based on PCA-based clusters (Figure S4). The two sets of stratified analyses were qualitatively similar overall. In particular, we observed very similar profiles of statistical significance for the Mexican PCA-based cluster as compared to the self-reported group of Mexican origin. This is not surprising because there was strong correspondence between individuals classified as Mexican by PCA-based cluster versus self-report. In analysis of individuals with self-reported origin in the Dominican Republic, we also see a suggestion of association in the vicinity of the index SNP rs325, but no SNPs reach the Bonferroni threshold for statistical significance. We did not observe any other statistically significant or suggestive signals of genetic association in stratified analysis of those with country/region of origin self-reported as Central America, Cuba, Puerto Rico or South America.

### Genetic association analysis of SNPs in the TRIB1 gene region

We selected 45 SNPs (17 genotyped and 28 imputed) that exhibited modest to strong linkage disequilibrium (LD) with the *TRIB1* index SNPs in Caucasians. We then performed genetic association analysis for these 45 SNPs, both pooled across the entire MESA Hispanic cohort and stratified by PCA-based Hispanic subgroup.

**Figure 4:**
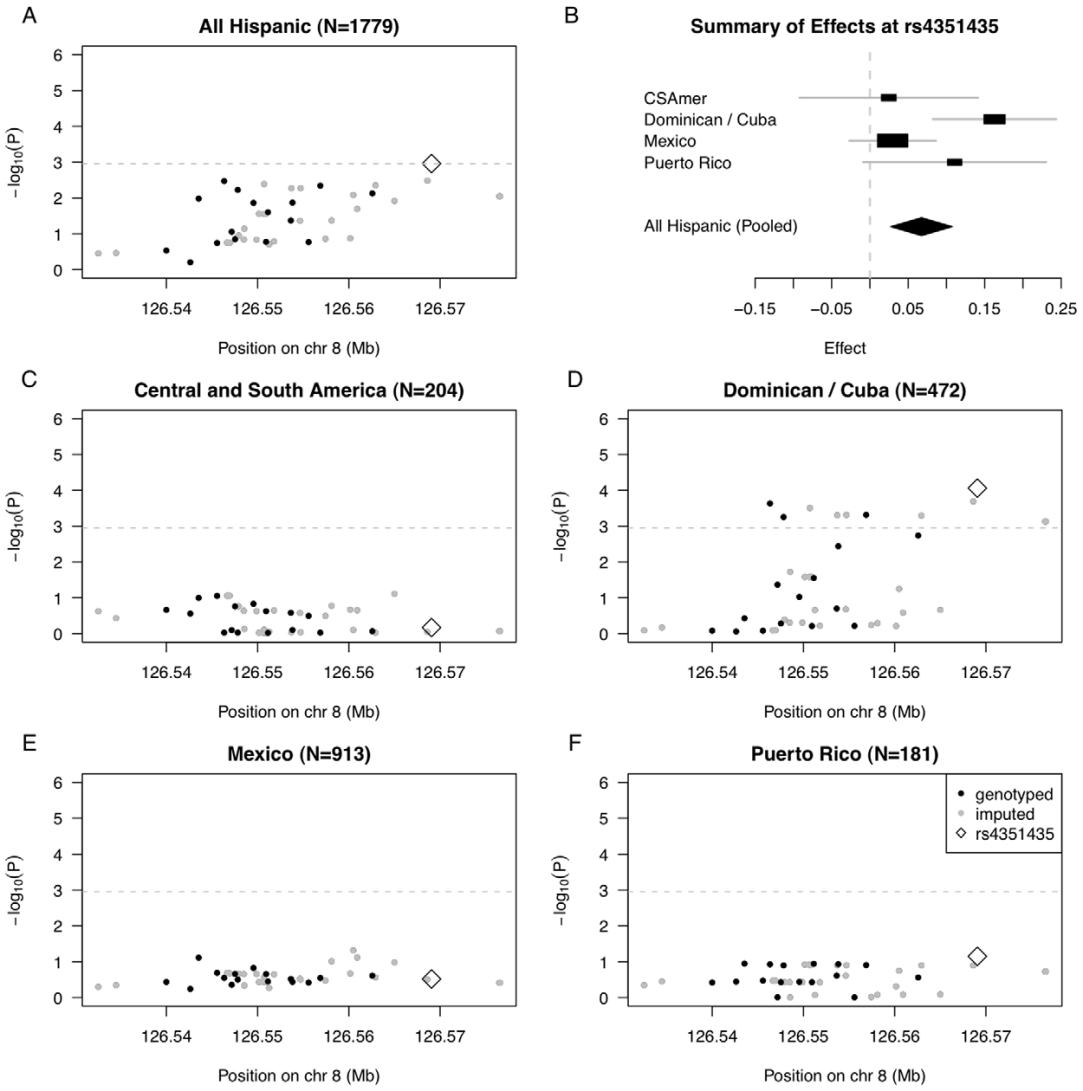
Summary of regional association for SNPs in the *TRIB1* gene region with triglycerides (modeled on a log scale). (A) Strength of association versus SNP position on chromosome 8 based on pooled analysis of MESA Hispanic individuals; (B) Forest plot of effects (with 95% CIs) reported in subsets of the MESA Hispanic cohort, using subgroups obtained from PCA-based cluster-analysis; and Strength of association versus SNP position on chromosome 8 based on stratified analysis of inferred clusters corresponding to (C) Central/South America, (D) the Dominican Republic and Cuba, (E) Mexico, and (F) Puerto Rico. In plots (A) and (C–F), genotyped SNPs are indicated as solid black dots, imputed SNPs as solid gray dots, the genotyped SNP rs4351435 as an open black diamond, and horizontal dashed gray lines indicate a conservative Bonferroni-threshold for statistical significance based on multiple testing of 45 SNPs.

In pooled analysis of the MESA Hispanic cohort, rs4351435 (*P* = 1.09E-3) is the only SNP that reaches the Bonferroni cutoff (0.05/45 SNPs = 1.11E-3) (Figure 4A, Table S7). In stratified analysis of the most strongly associated SNP rs4351435, we find the Dominican/Cuban subgroup has the strongest estimated effect of 0.163 (SE = 0.041, coded allele freq. 0.213) on log triglycerides (log mg/dL) per copy of the coded G allele, followed by the Puerto Rican subgroup with an estimated effect of 0.111 (SE = 0.061, coded allele freq. 0.203). Estimated effects for the Central/South American and Mexican subgroups are considerably closer to zero, at 0.025 (SE = 0.059, coded allele freq. 0.203) and 0.030 (SE = 0.029, coded allele freq. 0.235), respectively (Figure 4B), and a test of heterogeneity in genetic effects across the four subgroups is statistically significant (*P* = 0.044, heterogeneity I^2^ = 38.1).

The observed differences in genetic effects across the four Hispanic subgroups suggest the strength of genetic association increases with the proportion of African ancestry, seen in higher proportions for the Dominican/Cuban and Puerto Rican subgroups compared to the Central/South American and Mexican subgroups. To quantify this relationship, we added an interaction between the SNP rs4351435 and PC1 in the linear model used to assess genetic association in the pooled Hispanic cohort. The rs4351435-PC1 interaction term is statistically significant (P = 0.019), suggesting heterogeneity in effects of rs4351435 on triglycerides is attributable in part to the proportion of African versus Native American or Caucasian ancestry (as quantified by PC1) within the MESA Hispanic cohort. In validation, we observed statistically significant association of the rs4351435 SNP with triglycerides in analysis 2,067 individuals from the MESA African American cohort (*P* = 0.037). Interestingly, the index SNP rs2954029 originally identified in studies of Caucasians was neither statistically significant in association analysis of the pooled MESA Hispanic cohort (*P* = 0.134) nor in analysis of the MESA African American cohort (*P* = 0.748). These results suggest that while the *TRIB1* gene plays a role in determining triglycerides in Caucasians as well as African American and Dominican/Cuban individuals, the variants underlying this association vary by genetic ancestry. Another possibility is that the SNP effects interact with an environmental or dietary factor that is correlated with proportion of African ancestry within the MESA Hispanic cohort.

We went on to examine genetic association in stratified analysis of the four Hispanic subgroups for the full set of 45 *TRIB1* SNPs (Figure 4C–4F; Tables S8, S9, S10, S11). While there was only one statistically significant SNP reaching the Bonferroni threshold in pooled analysis of the full MESA Hispanic cohort, we observe 11 SNPs reaching statistical significance in stratified analysis of the Dominican and Cuban subgroup. Further, the p-value of the most strongly associated SNP rs4351435 is more than ten times stronger in stratified analysis of the Dominican and Cuban subgroup (*P* = 8.67E-5) as compared to pooled analysis (*P* = 1.09E-3). We do not observe any SNPs reaching the threshold for statistical significance in analysis of the Central/South American, Mexican, or Puerto Rican subgroups. These results indicate that when there is considerable heterogeneity in genetic effects observed across the full Hispanic cohort, stratified analysis may provide better resolution to uncover genetic association signals that exhibit stronger effects within a single subgroup of the Hispanic cohort.

As we did for the genetic association analysis of *LPL*, we performed genetic association analyses of *TRIB1* stratified by self-reported country/region of origin to compare with results of analyses stratified by PCA-based clusters (Figure S5). As we saw for *LPL*, the results of genetic association analysis were similar for the two sets of analyses. Stratification based on self-reported country/region of origin did produce generally weaker profiles of statistical significance, partially due to grouping by stratum with fewer individuals. Thus, genetic association analysis among those with self-reported country/region of origin in the Dominican Republic (Figure S5C) produces suggestive evidence of association, but does not reach the statistically significant result seen in analysis of the Dominican/Cuban PCA-based cluster (Figure 4D).

## Discussion

Our detailed population structure analysis of 1,374 unrelated individuals from the MESA Hispanic cohort, with reference to HapMap phase III and HGDP samples, provides a comprehensive view of the complex population structure inherent to the MESA Hispanic cohort. Our analyses document contributions of Caucasian, African and Native American ancestry to present day U.S. Hispanic populations. Our results are consistent with historical records and with previous studies [1], including an analysis of 705 Hispanic individuals from the MESA cohort using 199 AIMs [3]. Drawing on the resolution of the genome-wide genotype data recently available for the full MESA cohort through MESA SHARe (including 1,374 unrelated individuals and 2,174 Hispanic individuals in total), as well as geographic diversity of the MESA cohort with regard to Hispanic country/region of origin, we demonstrate diversity among the Native American ancestral populations contributing to present day Hispanic populations, consistent with Latin American historical records. In particular, we find the third and fourth principal components (PCs) of ancestry bring out a striking South-to-North axis in the available Native American samples that clearly separates Mexican versus Central/South American samples in MESA. Further, we find the fourth PC of ancestry separates Puerto Ricans from all other Hispanic groups in MESA, although there are no appropriate Native American samples available to verify this axis aligns with genetic differences in the corresponding Native American founders. To our knowledge, this is the first time diversity in underlying sources of Native American ancestry has been documented at this level of resolution, and in a sample reflecting the broad diversity of Hispanic origins represented among U.S. Hispanics.

Our population structure analysis and subsequent cluster analysis identified at least four distinct groups within the surveyed Hispanic cohort. Although self-identified country/region of origin was not used to inform the cluster analysis, the resulting groups showed remarkably close agreement with self-identification data, allowing us to identify the resulting PCA-based clusters roughly with the following four regions: Central/South America, the Dominican Republic and Cuba, Mexico, and Puerto Rico. We emphasize that the labels we have assigned to these clusters should be regarded loosely, provided as an aid to interpretation of results, but not intended as a vast generalization of individuals from the said regions. Indeed, we recognize there is great diversity in genetic ancestry within each of these regions, and this diversity is documented extensively in our population structure analysis. Taken as a whole, our thorough population structure analysis and genetic analysis brings forth the important message that the “Hispanic” population is in fact highly heterogeneous and genetically diverse. Our thorough genetic population structure analysis reveals genetic subgroups that correspond with groups of Hispanics with shared culture and history.

One notable difference between our study and previous reports of population structure in Hispanic groups (*e.g.* Bryc *et al.* [1]) lies in how we incorporate information from external reference panels. While previous studies performed population structure analysis on pooled data sets including Hispanic samples and relevant individuals from the HapMap, HGDP or other reference panels [1], [2], [4], we compute principal components in MESA Hispanic samples alone, leveraging information from the reference panels by projecting these principal components across samples. For the purpose of understanding the Hispanic population, we find it is more informative to focus the analysis in this way, particularly for characterizing finer structure within Native American ancestral groups. Our focused approach to population structure analysis is feasible mainly because our sample size is considerably larger than that available to previous studies of Hispanic population structure.

To demonstrate the differences described above, we performed principal component analysis for unrelated individuals from the MESA Hispanic cohort pooled with samples from the HapMap and HGDP (Figures S6 and S7). For the first two PCs, the results of the our pooled PCA as well as that of Bryc *et al.* [1] agree largely with those seen in our PCA computed for MESA Hispanic samples only. For higher order PCs, we do see qualitative differences in pooled versus focused PCA. Notably, there is a clear separation of Puerto Rican samples from Central and South American samples in the plot of PC4 versus PC3 from analysis of the MESA Hispanic cohort alone (Figure 1), but this separation is not observed in higher order PCs from our pooled PCA (Figure S7) nor is it apparent in higher order PCs from pooled analysis presented in Bryc *et al.* [1]. This comparison further indicates the finer differences we detected among Hispanic and Native American groups of distinct geographic origins were possible due to our focused approach of computing principal components using genotype data from the MESA Hispanic cohort only.

There are several immediate applications of our work for genetic analysis of Hispanic cohorts. We have defined at least four distinct clusters of genetic ancestry within the MESA Hispanic cohort, and we suggest future genetic analyses of MESA Hispanics should be stratified across these clusters. Of course, stratified analysis will introduce problems of multiple testing, and reduced sample sizes within strata. When the number of individuals with phenotypes available does not allow stratification, a reasonable approach will be to perform pooled analysis of the entire Hispanic cohort, with adjustment for the strongest principal components of ancestry. An intermediate approach will be to stratify using just two clusters inferred from the first two principal components of ancestry.

To demonstrate our recommendations for genetic association analysis taking into account our documented genetic diversity in the MESA Hispanic cohort, we performed association analysis of triglycerides with SNPs in the *LPL* and *TRIB1* gene regions, previously implicated in GWAS of Caucasians but unconfirmed in Hispanics. We began with pooled analysis, in which we found SNPs reaching the Bonferroni thresholds for statistical significance in each of the two gene regions. Follow-up by stratified analysis in each of four subgroups of the MESA Hispanic cohort revealed a suggestion of heterogeneity in the strongest functional *LPL* variant rs328 (Ser474Stop) and statistically significant evidence for genetic heterogeneity at the most strongly associated *TRIB1* SNP rs4351435. Furthermore, evidence for the *TRIB1* SNP rs4351435 was substantially stronger in stratified analysis of the Dominican and Cuban subgroup alone, as compared to pooled analysis of the full MESA Hispanic cohort. Our genetic association analyses indicate pooled analysis provides good power to detect variants exhibiting little heterogeneity in genetic effects, while stratified analysis will provide an advantage in detecting SNPs with heterogeneity in which the genetic effect is strong for one subgroup and close to zero in other subgroups of the Hispanic cohort.

In practice, whether a formal test of heterogeneity is statistically significant or not, examining heterogeneity by effect plots or other tools will be an important step toward identifying the most promising samples for follow-up and replication studies. We do not expect genetic diversity will be the sole cause of heterogeneity in SNP effects. Evidence both from our current work and from previous studies [13] indicates that genetic distance correlates with geography, that geography correlates to a certain extent with environmental exposures as well as with social and cultural factors [21], and that these factors, in turn, may serve as independent predictors of cardiovascular outcomes of interest or modifiers of genetic effects [21], [22], [23], [24]. Given the strong correspondence between our inferred genetic clusters and self-identified country/region of origin, stratified analysis will serve as a general strategy to examine differences across subgroups of the Hispanic cohort, which differ not only in genetic origins but also in terms of lifestyle factors (*e,g,* diet) as well as other social and cultural factors associated with diverse regions of origin and diverse histories of migration to the United States.

Toward generalizing our results to the United States Hispanic population as a whole, it is important to keep in mind the demographics of our cohort. The MESA Hispanic cohort represent primarily recent immigrants to the United States, with 65% born outside the United States, and another 28% having at least one parent born outside the United States [25]. Our reported population structure analyses may be biased in part by this distribution of immigration to the United States, and it is possible that self-reported Hispanics whose families have been in the United States for multiple generations may exhibit different patterns of ancestry, including a greater degree of admixture across the Hispanic countries/regions of origin represented in this study, as well as admixture with other racial/ethnic groups living in the United States (*e.g.* Caucasian, Asian, or African American).

Individuals in the MESA Hispanic cohort were recruited primarily from three sites in the United States, namely New York City, Minneapolis, and Los Angeles. Based on this geographic distribution, the MESA Hispanic cohort cannot be regarded as a fully representative sample of Hispanics from across the United States. Examining the self-identification data for an unrelated subset of 1,374 individuals from the MESA Hispanic cohort (51.5% Mexican, 14.0% Puerto Rican, 3.6% Cuban, 14.8% Central/South American, 14.8% Dominican, and 1.2% other/not specified) compared to the U.S. Hispanic population (63.0% Mexican, 9.2% Puerto Rican, 3.5% Cuban, 13.4% Central/South American, 2.8% Dominican, and 8.1% other, based on data from the United States 2010 Census [26]), we find generally good agreement between countries/regions of origin in the MESA Hispanic cohort compared to the U.S. Hispanic population. The notably higher representation of individuals with Dominican origin in the MESA Hispanic cohort reflects the fact that New York City, an area with one of the highest concentrations of Dominicans in the United States, was one of the main recruitment sites for MESA Hispanic participants (Table S12). Overall, these data suggest the MESA Hispanic cohort does provide good representation of the major countries/regions of Hispanic origin found in the U.S. Hispanic population. Thus, the population structure analysis of the MESA Hispanic cohort will provide a valuable resource toward understanding genetic diversity in the broader U.S. Hispanic population. However, given the possibility of migration bias due in part to socioeconomic or cultural factors, we caution against drawing on our results to interpret genetic diversity of Hispanics living outside the United States.

## Methods

### Ethics statement

All MESA participants gave written informed consent, including consent to participate in genetic studies. This MESA study was conducted under Institutional Review Board approval at all study sites, including the Cedars-Sinai Medical Center and the University of Virginia.

### Genotype data

The Multi-Ethnic Study of Atherosclerosis (MESA) is a longitudinal study of subclinical cardiovascular disease and risk factors that predict progression to clinically overt cardiovascular disease or progression of the subclinical disease [27]. The first clinic visits occurred in 2000 in 6,814 participants recruited from six field centers across the United States. Approximately 38% of the recruited participants are White, 28% African-American, 22% Hispanic, and 12% Asian, predominantly of Chinese descent. Genome-wide genotyping was performed in 2009 using the Affymetrix Human SNP array 6.0. SNPs were filtered for SNP level call rate <95% and individual level call rate <95%, and monomorphic SNPs were removed. Examining the distribution of heterozygosity rates across all genotyped SNPs, we observed a generally uniform distribution between 0–53%, with less than 0.01% of SNPs having heterozygosity >53%. Thus, we removed all SNPs with heterozygosity >53%. The cleaned genotypic data was deposited with MESA phenotypic data into dbGaP as the MESA SHARe project (study accession phs000209, http://www.ncbi.nlm.nih.gov/projects/gap/cgi-bin/study.cgi?study_id=phs000209.v4.p1) for 8,227 individuals (2,686 Caucasian, 777 Chinese, 2,590 non-Hispanic African-American, and 2,174 Hispanic) with 897,981 SNPs passing study specific quality control (QC). Due to differences in allele frequencies across the MESA ethnic groups, there was no filter of minor allele frequency prior to release of the genotype data on dbGaP. Thus, we applied a filter on minor allele frequency at the stage of genetic association analysis (see “*Selection of SNPs for genetic association analysis*” below).

The country or region of Hispanic origin was coded for individuals in the MESA Hispanic cohort using the following categories: Mexican, Dominican, Puerto Rican, Cuban, Central American, South American or other Hispanic subgroup. Participant self-identification was available for 83% of individuals in the MESA Hispanic cohort. For the remaining 17% where this self-identification was not provided, the data were obtained from the place of birth of the most recent generation (among the participant, parents, and grandparents) outside of the 50 United States as follows:

1. If the participant reported place of birth outside of the United States, Europe, and Asia, then that place of birth was used.
2. Otherwise, if there was a single reported place of birth outside of the United States, Europe, and Asia for both parents, then that place of birth was used.
3. Otherwise, the place of birth of the grandparents was used. If more than one place of birth outside of the Unites States, Europe, and Asia was specified, then the majority was used.

To make use of the HapMap phase III release 3 genotypes as a reference panel for our analysis of MESA samples, we began with 1,397 individuals from the following 11 HapMap populations: ASW, CEU, CHB, CHD, GIH, JPT, LWK, MEX, MKK, TSI, and YRI. Genotype data were obtained by the HapMap 3 Consortium using the Affymetrix Human SNP array 6.0 and the Illumina Human1M-single beadchip. Following data merging and cleaning [9], there were 1,457,897 SNPs in the publicly available data downloaded from http://hapmap.ncbi.nlm.nih.gov/downloads/ in PLINK [28] format.

Publicly available genotype data for the Human Genome Diversity Project (HGDP) were downloaded from http://hagsc.org/hgdp/files.html for 1,043 individuals on 660,918 SNPs [11]. These data included genotypes generated on Illumina 650Y arrays, with a GenCall Score cutoff of 0.25. The publicly available genotypes were filtered on overall SNP level call rate <98.5%, with no additional filtering of SNPs.

### Data management and quality control

To allow common analysis across the three sources of genotype data (MESA, HapMap and HGDP), the first step was to merge the genotype files aligned on a common set of alleles. To avoid any ambiguity in strand alignment, we merged the genotype data files using an approach that does not rely on a priori knowledge of strand direction in annotation files. Briefly, SNPs with alleles A/G or C/T could be merged across genotype files, unambiguously flipping alleles (A→T, G→C) as necessary. The small proportion of SNPs with alleles A/T or G/C could not be merged using this method, due to ambiguities in strand-flipping, as so were removed. Although this strand-flipping procedure forces us to remove the small proportion of ambiguous SNPs, the resulting set of retained SNPs is less error prone (in terms of called alleles) than if we had relied on strand direction reported in annotation files alone. The allele flipping procedure described as run as currently implemented in the software package KING [29].

After file merging and allele flipping, we filtered on SNP level call rate <95% across 12,058 genotyped individuals from the three genotype data sets (MESA, HapMap and HGDP), resulting in 144,564 autosomal SNPs common to all data sets. We then filtered on individual level call rate <95%, resulting in a combined set of 10,666 individuals across the three genotype data sets with overall genotyping rate 0.998 across the 144,564 autosomal SNPs.

### Relationship inference

To identify an unrelated set of MESA individuals for population structure analysis, we performed relationship inference using a method we developed recently for accurate relationship up to the 3^rd^-degree using genotypes from genome-wide association data, implemented in the freely available software package KING [29]. Because precision of the method increases with the number of typed SNPs available for any pair of individuals, relationship inference was performed using the full set of SNPs, prior to filter for SNPs common to all three genotype data sets. Individuals were clustered into connected groups (i.e. families) using the KING option “cluster –3”, which defines clusters such that any pair of individuals with inferred relationship as distant as 3^rd^-degree are grouped together.

A list of unrelated individuals from MESA was constructed by selecting one individual from each known family (common family ID reported in the downloaded data), and further thinning to include no more than one individual from each cluster inferred in the KING clustering of individuals with inferred relationships up to the 3^rd^ degree. While we recognize that some inferred relationships of the 3^rd^ degree may be false positives, we used this stringent criterion to ensure we had a clean set of individuals for population structure analysis. The final list of unrelated individuals generated by this procedure included 6,496 MESA participants. From this group of individuals, we further removed 5 Hispanic individuals identified as outliers according to principal components of ancestry, as computed in SMARTPCA (see “*Principal component analysis*” below).

Because the HapMap samples were collected with systematic relatedness including father-mother-child trios [9], we implemented an algorithm using the software KING [29] to extract multiple unrelated individuals from a pedigree, when available. The algorithm, available using the KING option “–unrelated” proceeds as follows. Related individuals (defined by existing pedigree or estimated kinship coefficient <0.088) are first clustered into connected groups (i.e., families). Within each family cluster, individuals are ranked according to the count of unrelated family members (having estimated kinship coefficient <0.022). To construct a set of unrelated individuals, we first select the individual with the largest count of unrelated individuals within the family cluster. We then proceed to choose the individual with the next highest rank (number of unrelated family members) within the family cluster, only if that individual is not related to any of the previously selected individuals in the list of unrelated individuals. We applied this algorithm to construct a set of 1,096 individuals from the HapMap and 922 individuals from the HGDP, with no 1^st^- or 2^nd^-degree relatives in the unrelated set.

Details of the data set after SNP QC, individual-level genotype QC, by cohort representation and status of inclusion in the final set of unrelated individuals, are provided in Table S1.

### Constructing a minimal LD set of SNPs

Prior to population structure analysis, we first constructed a subset of typed SNPs, thinned for linkage disequilibrium (LD) among MESA samples self-identified as Hispanic. Based on the assumption that Hispanics have a considerable proportion of Caucasian ancestry, we first removed from consideration SNPs in regions of known long-range linkage disequilibrium among Caucasians [30], including the HLA region (Chr 6: 24.5–34.5 Mb), a chromosome 8 inversion (Chr 8: 113–116 Mb), and a region on chromosome 11 (Chr 11: 45–58 Mb). We then thinned for local LD within an unrelated subset of the MESA Hispanic cohort using the PLINK [28] option “–indep-pairwise” to create a subset of typed SNPs thinned for pairwise R-squared no more than 0.2 in a 100 SNP window, moving the windows 25 SNPs at a time. This LD pruning procedure resulted in a set of 64,199 autosomal SNPs of minimal LD among the MESA Hispanic samples that are the focus of the current study. The resulting SNP set provides good resolution for global ancestry inference by both unsupervised (principal component analysis) and supervised (model-based clustering, as in the program STRUCTURE [8]) analysis.

### Principal component analysis

Using our LD-thinned subset of 64,199 SNPs, we performed Principal Component Analysis (PCA) as implemented in the program SMARTPCA [5], [6] from the software package EIGENSTRAT to compute principal components (PCs) of ancestry for an unrelated subset of 1,374 self-reported Hispanic individuals from MESA. In computing the PCs, we performed additional LD correction by using results of regression on the previous 5 SNPs as input to the PCA (SMARTPCA option “nsnpldregress”), and performed 5 iterations of outlier removal in which we removed individuals with computed values more than 10 standard deviations from mean along along the first 6 PCs of ancestry. Based on this procedure, five outliers were removed from an initial set of 1,379 unrelated MESA Hispanic individuals, prior to computation of the final set of PCs with 1,374 unrelated individuals.

Following computation of the PCs using the unrelated subset of MESA Hispanic individuals, we used SMARTPCA to project these components to all MESA samples that were not included in the PCA (including non-Hispanic samples), as well as the HapMap and HGDP samples, to assist in interpretation of the strongest PCs (corresponding to the largest eigenvalues).

As principal component analysis is known to be sensitive to outliers and undetected family structure, both of which can produce spurious PCs, we undertook a series of QC steps to assess properties of the components reported by SMARTPCA. First, we constructed histograms and QQ-plots to assess symmetry and normality of the distribution of loadings for each principal component. We found the distributions of loadings for the first four PCs closely matched the ideal symmetric, standard normal distribution, with no coefficients more extreme than 4.6. For higher PCs, we observed loadings as extreme as 6.4 (the loadings should follow a standard normal distribution). We also performed genome-wide association of each principal component as a quantitative trait among MESA Hispanics, using a method that accounts for familial correlation [31] as implemented in the software GDT [32], to assess the extent to which the component serves as a marker of genome-wide population stratification, versus strong correlation with smaller chromosomal regions, as would occur if the component was produced as a result of long-range LD. Individuals with principal component values greater than three standard deviations from the mean were removed prior to analysis of each principal component. We did not observe any principal components that appeared to reflect influence of long-range LD.

For comparison with our PCA of the MESA Hispanic cohort with projection to relevant reference panels, we performed PCA for unrelated individuals from the MESA Hispanic cohort pooled with samples from the HapMap and HGDP, using the same set of 64,199 SNPs selected for population structure analysis performed on the MESA Hispanic cohort. The resulting principal components from pooled analysis were subject to the same QC steps as applied for the MESA Hispanic-specific analysis, including examination of the distributions of PC loadings, and genome-wide association analysis of each PC as a quantitative trait to assess potential effects of long range LD.

### Model-based cluster analysis

We performed model-based cluster analysis using our LD-thinned subset of 64,199 SNPs using the package ADMIXTURE [7]. This software package implements the same clustering method as in STRUCTURE [8], using a block relaxation approach implemented with a novel quasi-Newton acceleration method that makes the method computationally feasible for much larger data sets [7], both in terms of the number of individuals and the number of SNPs.

In order to obtain a clear characterization of the MESA Hispanic cohort, we first performed the ADMIXTURE analysis for the same unrelated subset of 1,374 MESA Hispanic individuals that we used to perform PCA. We ran this analysis for K values 1, …, 10, assessing results for each of these runs in terms of cross validation error, as well as with graphic displays of proportions of ancestry across self-identified Hispanic country/region of origin.

### k-means cluster analysis to infer Hispanic subgroups

Visual inspection of the first four PCs of ancestry (Figure 1) suggested at least four distinct groups of individuals defined by these PCs, roughly divided along PC1 and PC4, with PC2 and PC3 reflecting variation within those groups. We have not ruled out the possibility of further substructure beyond these four visually discernable clusters. However, we choose to limit the number of clusters to four, based on the practical consideration that a larger number of clusters would lead to within-cluster sample sizes too small to allow subsequent genetic association analyses to be stratified by cluster.

We performed k-means clustering in the statistical software R [33], using the first four principal components of ancestry to define four major groups (k = 4) within the MESA Hispanic cohort. Starting values for cluster centers were assigned based on means observed within the upper and lower strata of values for PC1 and PC4. Based on overall correspondence between the cluster assignments and self-identified country/region of origin (Table 2), we labeled the four subgroups resulting from k-means clustering as Central/South American, Dominican, Mexican and Puerto Rican. Cluster assignments were made for all N = 2,169 individuals from the MESA Hispanic cohort with computed PCs available (5 individuals were excluded because they were removed as outliers during computation of PCs).

### Selection of SNPs for genetic association analysis

To assess association of SNPs in the *LPL* gene region with triglycerides, we began by selecting SNPs of interest in the region, with a focus on the index SNPs rs12678919 and rs10096633 reported in previous GWAS of Caucasians [14], [15], [16], [17], [18]. Due to patterns of linkage disequilibrium, the index SNPs identified in previous studies of Caucasians are not necessarily causal in determining genetic association with the phenotype of interest. However, we do expect the index SNPs are in linkage disequilibrium with the putative causal variant(s) underlying the genetic association. To improve our chance of capturing the causal variant(s) in our association analysis, we expanded our SNP set to include any SNPs exhibiting strong pairwise LD with our two initial SNPs (R-squared >0.7 in the HapMap II+III CEU samples, using release 28, NCBI Build 36 (dbSNP b126)). Of these 33 SNPs, 8 were genotyped on Affy 6.0 and passed genotype QC. IMPUTE version 2.1.0 was used to perform imputation for the MESA SHARe Hispanic participants (chromosomes 1–22) using HapMap Phase I and II - CEU+YRI+CHB+JPT as the reference panel (release #22 - NCBI Build 36 (dbSNP b126)), and another 25 could be imputed with quality >0.8, based on the observed versus expected variance quality metric [34]. We verified the minor allele frequency of all these SNPs was greater than 0.01 in the pooled MESA Hispanic cohort, as well as in all stratified analyses. In this way, we identified 33 SNPs to be included in a more comprehensive analysis of the *LPL* gene region.

For selection of SNPs to be included in association analysis of the *TRIB1* gene region, we used a strategy similar to that for *LPL*. We began by targeting the SNP rs2954029 previously reported in GWAS of Caucasians [14], [15], [18]. We further selected 19 SNPs exhibiting strong pairwise LD with our initial index SNP (R-squared >0.7 in the HapMap II+III CEU samples, using release 28, NCBI Build 36 (dbSNP b126)). Association analysis of these 19 SNPs did not reveal any results approaching statistical significance, so we expanded the association analysis to 45 SNPs having modest to strong LD with the rs2954029 index SNP (R-squared >0.3 in the HapMap II+III CEU samples). Of these 45 SNPs, 17 were genotyped on Affy 6.0 and passed genotype QC, and the other 28 could be imputed with quality >0.8, based on the observed versus expected variance quality metric [34].

### Genetic association analysis for fasting triglycerides

Fasting triglycerides were measured in plasma using a glycerol blanked enzymatic method (Trig/GB, Roche Diagnostics, Indianapolis, Indiana). To select individuals to be included in this analysis, we began with the full set of N = 2,169 individuals with data available from principal component analysis. We then restricted the data set to individuals with triglyceride phenotypes available (N = 2,151) and no known use of any lipid lowering medication (N = 1,788). To allow study site to be included as a covariate in genetic association analysis, we restricted the data set to individuals from study sites with data available for at least 20 individuals (N = 1,782). Outliers were defined as individuals with log triglyceride values more then 3.5 SD from the mean, with the mean and SD calculated separately for each of the five analyses performed (pooled analysis of all MESA Hispanics, and stratified analysis of the four subgroups).

Based on these criteria, we performed pooled analysis of all MESA Hispanics (N = 1,779), as well as stratified analysis within the PCA-based clusters corresponding to Central and South America (N = 204), the Dominican Republic and Cuba (N = 472), Mexico (N = 913) and Puerto Rico (N = 181). For comparison, we also performed stratified analysis by self-reported country/region of origin for the following groups: Central America (N = 109), Cuba (N = 34), the Dominican Republic (N = 315), Mexico (N = 961), Puerto Rico (N = 202), and South America (N = 123). Analysis was performed using an additive model with a linear mixed-effects model to account for familial relationships as implemented in the package R/GWAF [19]. We used a basic model including the covariates gender, age, study site, and the first four PCs of ancestry, using principal components computed for the full Hispanic cohort. PC2 was not included in stratified analyses of Dominican/Cuban, Mexican and Puerto Rican PCA-based clusters, for which we observed the correlation between PC1 and PC2 was −0.95, 0.90, and −0.89, respectively. Similarly, PC2 was omitted from stratified analyses with country/region of origin self-reported as Cuba, the Dominican Republic, Mexico, and Puerto Rico, for which we observed the correlation between PC1 and PC2 was −0.94, −0.98, 0.89, and −0.89, respectively.

To assess genetic heterogeneity seen in stratified analysis of the four Hispanic subgroups, we performed a test of heterogeneity using Cochran's Q and also examined the inconsistency metric I^2^ which quantifies the proportion of total variation across studies due to heterogeneity rather than chance [35].

To validate results seen for SNPs exhibiting the strongest association in the Hispanic cohort, we performed genetic association analysis in the MESA African American cohort. We began with the full set of N = 2,588 consenting individuals from MESA or MESA Family self-identified as African American. There were N = 2,580 individual remaining after removing outliers from principal component analysis. We then restricted the data set to individuals with triglyceride phenotypes (N = 2,552) available and no known use of any lipid lowering medication (N = 2,071). All study sites had data available for at least 20 African American individuals. After removing outliers were defined as individuals with log triglyceride values more then 3.5 SD from the mean, we performed genetic association analysis of N = 2,067 individuals, using a linear mixed-effects model to account for familial relationships [19] and a basic model including the covariates gender, age, study site, and the first principal component of ancestry.

## Supporting Information

Figure S1 Top eight principal components of ancestry computed in an unrelated subset of 1,374 MESA Hispanic individuals, with projection to an unrelated subset of the remaining MESA samples. Individuals are labeled according to group inclusion as indicated.
(PDF)

Figure S2 Top eight principal components of ancestry computed in an unrelated subset of 1,374 MESA Hispanic individuals, with projection to an unrelated subset of individuals from the other MESA ethnic groups, in addition to samples from the HapMap and HGDP. Individuals are labeled according to group inclusion: “MESAHispNOS”="MESA Hispanic, Other or Unspecified country/region or origin”, other labels are self-explanatory. “HapHGDPAfrica” includes LWK and YRI from the HapMap, as well as African samples from the HGDP. “HapHGDPEurope” includes CEU and TSI from the HapMap and European samples from the HGDP. “HapHGDPCSAsia” includes GIH from the HapMap and Central/South Asian samples from the HGDP. “HapHGDPEastAsia” includes CHB, CHD, and JPT from the HapMap and East Asian samples from the HGDP. All other labels are self-explanatory.
(PDF)

Figure S3 Individual-level proportion of ancestry estimates from model-based clustering analysis in ADMIXTURE for 1,374 unrelated individuals of self-reported Hispanic origin from the Multi-Ethnic Study of Atherosclerosis (MESA), shown for K values 2 through 7.
(PDF)

Figure S4 Summary of regional association for SNPs in the *LPL* gene region with triglycerides (modeled on a log scale). Strength of association versus SNP position on chromosome 8 based on stratified analyses for self-reported country/region of origin corresponding to (A) Central America, (B) Cuba, (C) the Dominican Republic, (D) Mexico, (E) Puerto Rico, and (F) South America. Genotyped SNPs are indicated as solid black dots, imputed SNPs as solid gray dots, the imputed SNP rs328 as an open gray diamond, and horizontal dashed gray lines indicate a conservative Bonferroni-threshold for statistical significance based on multiple testing of 33 SNPs.
(PDF)

Figure S5 Summary of regional association for SNPs in the *TRIB1* gene region with triglycerides (modeled on a log scale). Strength of association versus SNP position on chromosome 8 based on stratified analyses for self-reported country/region of origin corresponding to (A) Central America, (B) Cuba, (C) the Dominican Republic, (D) Mexico, (E) Puerto Rico, and (F) South America. Genotyped SNPs are indicated as solid black dots, imputed SNPs as solid gray dots, the genotyped SNP rs4351435 as an open black diamond, and horizontal dashed gray lines indicate a conservative Bonferroni-threshold for statistical significance based on multiple testing of 45 SNPs.
(PDF)

Figure S6 Top eight principal components of ancestry computed in pooled analysis of unrelated MESA Hispanic individuals together with HapMap and HGDP samples, with projection to an unrelated subset of individuals from the other MESA ethnic groups. Individuals are labeled according to group inclusion: “MESAHispNOS”="MESA Hispanic, Other or Unspecified country/region or origin”, other labels are self-explanatory. “HapHGDPAfrica” includes LWK and YRI from the HapMap, as well as African samples from the HGDP. “HapHGDPEurope” includes CEU and TSI from the HapMap and European samples from the HGDP. “HapHGDPCSAsia” includes GIH from the HapMap and Central/South Asian samples from the HGDP. “HapHGDPEastAsia” includes CHB, CHD, and JPT from the HapMap and East Asian samples from the HGDP. All other labels are self-explanatory.
(PDF)

Figure S7 Top eight principal components of ancestry computed in pooled analysis of unrelated MESA Hispanic individuals together with HapMap and HGDP samples. Results are displayed for an unrelated subset of individuals from the MESA Hispanic cohort and key reference populations from the HapMap and HGDP.
(PDF)

Table S1 Representation of worldwide populations in population structure analysis. Counts of unrelated individuals are reported after relationship inference for systematic removal of related individuals, and outlier removal based on principal components of ancestry, as detailed in the METHODS.
(XLS)

Table S2 Results for association analysis of 33 SNPs in the *LPL* gene region with triglycerides in the pooled MESA Hispanic cohort. Analysis was performed using an additive model with a linear mixed-effects model to account for familial relationships, and inclusion of basic covariates gender, age, study site, and PCs of ancestry.
(CSV)

Table S3 Results for association analysis of 33 SNPs in the *LPL* gene region with triglycerides in stratified analysis of the Central/South American subgroup. Analysis was performed using an additive model with a linear mixed-effects model to account for familial relationships, and inclusion of basic covariates gender, age, study site, and PCs of ancestry.
(CSV)

Table S4 Results for association analysis of 33 SNPs in the *LPL* gene region with triglycerides in stratified analysis of the Dominican and Cuban subgroup. Analysis was performed using an additive model with a linear mixed-effects model to account for familial relationships, and inclusion of basic covariates gender, age, study site, and PCs of ancestry.
(CSV)

Table S5 Results for association analysis of 33 SNPs in the *LPL* gene region with triglycerides in stratified analysis of the Mexican subgroup. Analysis was performed using an additive model with a linear mixed-effects model to account for familial relationships, and inclusion of basic covariates gender, age, study site, and PCs of ancestry.
(CSV)

Table S6 Results for association analysis of 33 SNPs in the *LPL* gene region with triglycerides in stratified analysis of the Puerto Rican subgroup. Analysis was performed using an additive model with a linear mixed-effects model to account for familial relationships, and inclusion of basic covariates gender, age, study site, and PCs of ancestry.
(CSV)

Table S7 Results for association analysis of 45 SNPs in the *TRIB1* gene region with triglycerides in the pooled MESA Hispanic cohort. Analysis was performed using an additive model with a linear mixed-effects model to account for familial relationships, and inclusion of basic covariates gender, age, study site, and PCs of ancestry.
(CSV)

Table S8 Results for association analysis of 45 SNPs in the *TRIB1* gene region with triglycerides in stratified analysis of the Central/South American subgroup. Analysis was performed using an additive model with a linear mixed-effects model to account for familial relationships, and inclusion of basic covariates gender, age, study site, and PCs of ancestry.
(CSV)

Table S9 Results for association analysis of 45 SNPs in the *TRIB1* gene region with triglycerides in stratified analysis of the Dominican and Cuban subgroup. Analysis was performed using an additive model with a linear mixed-effects model to account for familial relationships, and inclusion of basic covariates gender, age, study site, and PCs of ancestry.
(CSV)

Table S10 Results for association analysis of 45 SNPs in the *TRIB1* gene region with triglycerides in stratified analysis of the Mexican subgroup. Analysis was performed using an additive model with a linear mixed-effects model to account for familial relationships, and inclusion of basic covariates gender, age, study site, and PCs of ancestry.
(CSV)

Table S11 Results for association analysis of 45 SNPs in the *TRIB1* gene region with triglycerides in stratified analysis of the Puerto Rican subgroup. Analysis was performed using an additive model with a linear mixed-effects model to account for familial relationships, and inclusion of basic covariates gender, age, study site, and PCs of ancestry.
(CSV)

Table S12 Representation of study sites for the full set of 1,374 unrelated MESA Hispanic individuals used for principal component analysis.
(XLS)
