# Supplementary figures and images for: Population Structure of Hispanics in the United States: The Multi-Ethnic Study of Atherosclerosis
Source: PLoS Genet. 2012 Apr 12;8(4):e1002640. doi: 10.1371/journal.pgen.1002640 (PMC3325201; doi:10.1371/journal.pgen.1002640)

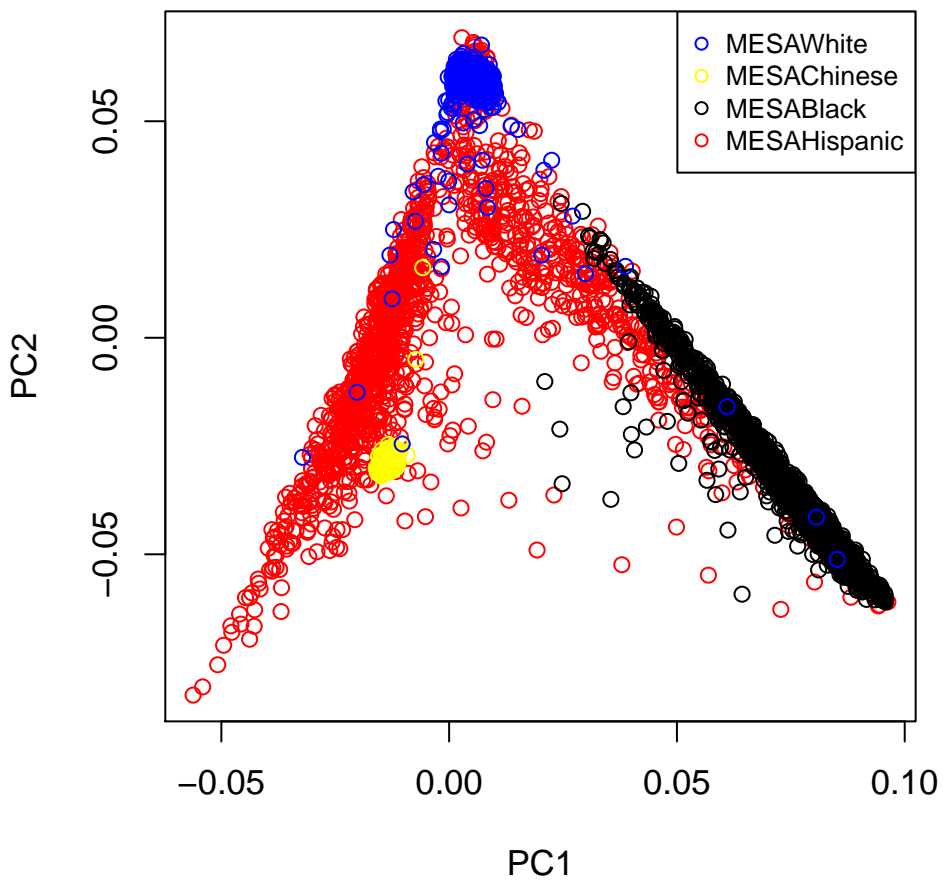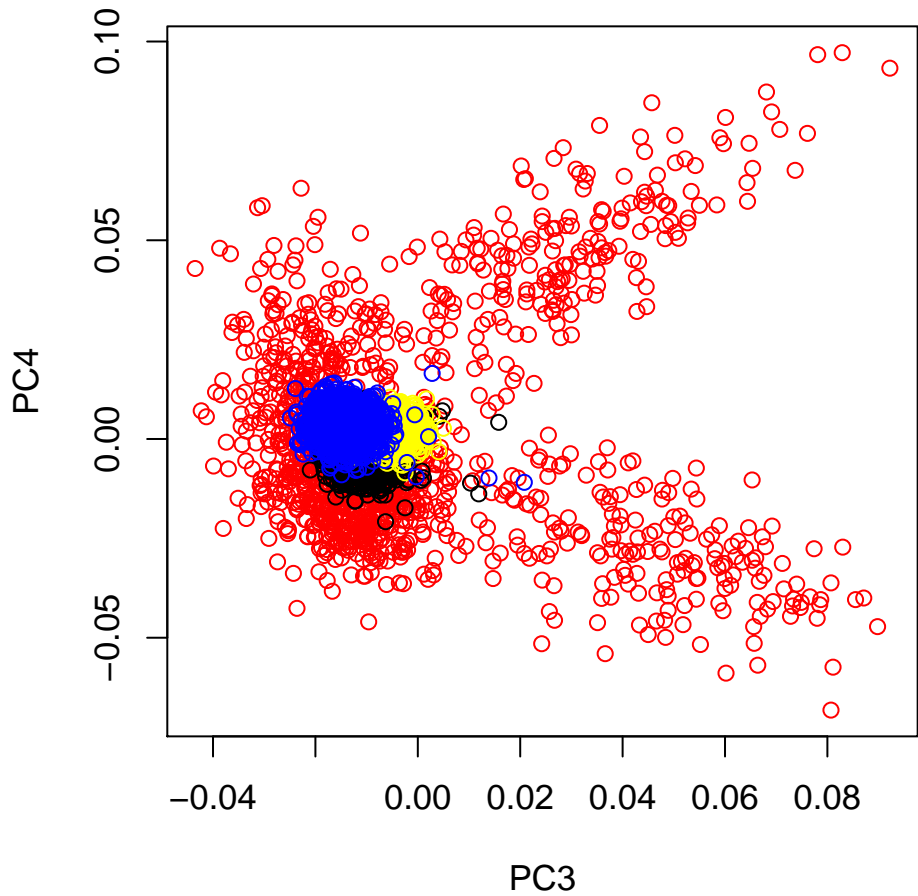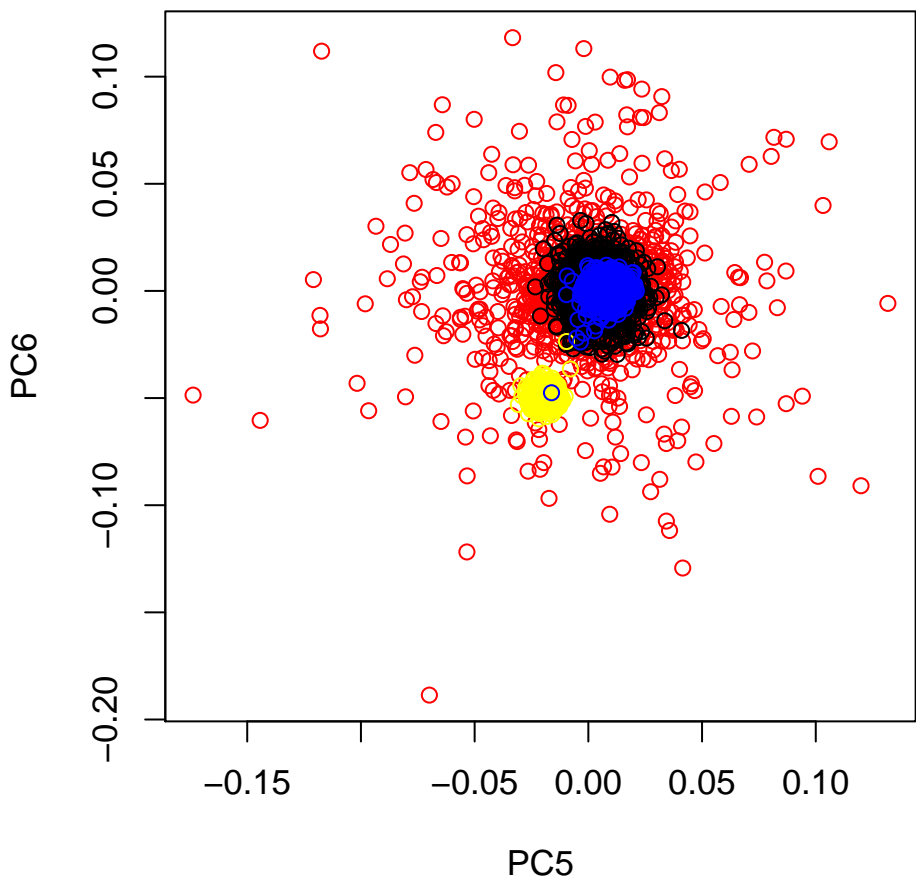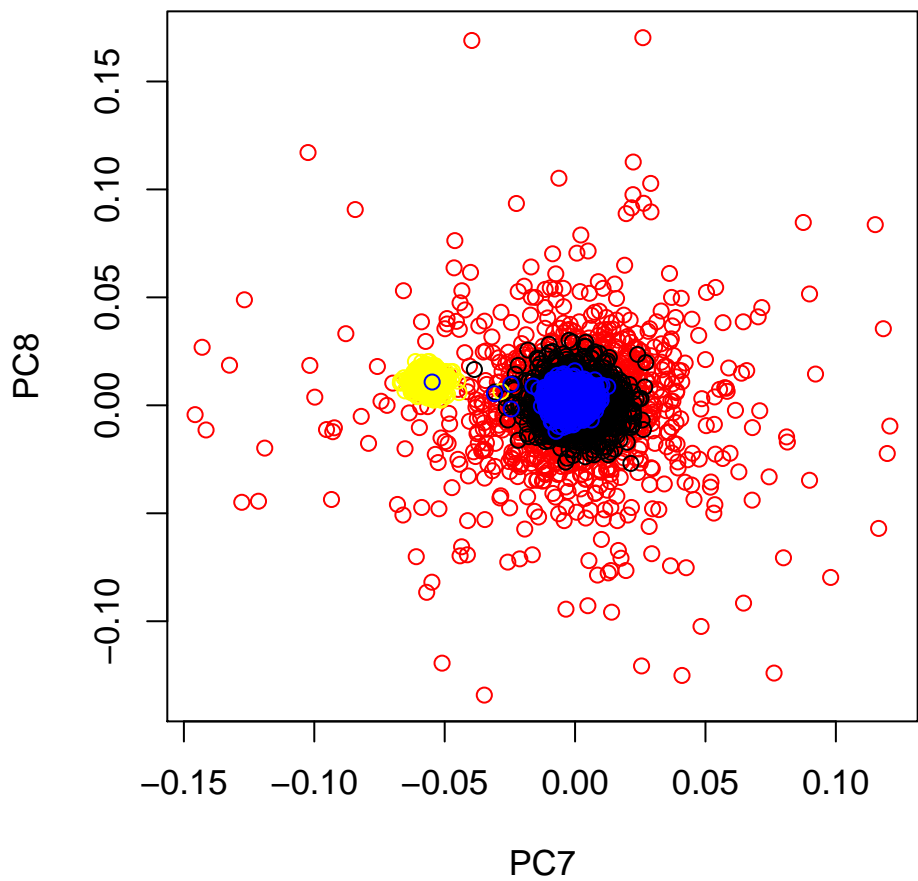

Supplement: Figure S1 — Top eight principal components of ancestry computed in an unrelated subset of 1,374 MESA Hispanic individuals, with projection to an unrelated subset of the remaining MESA samples. Individuals are labeled according to group inclusion as indicated. (PDF) [file pgen.1002640.s001.pdf]

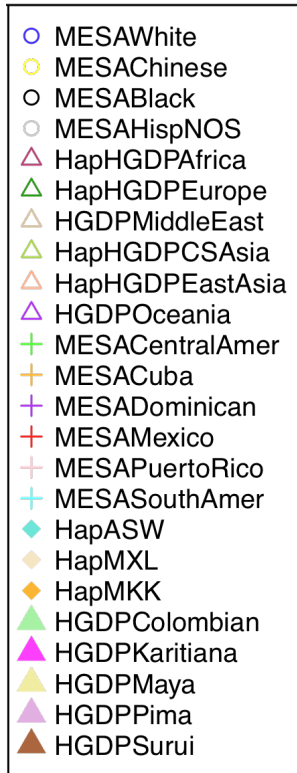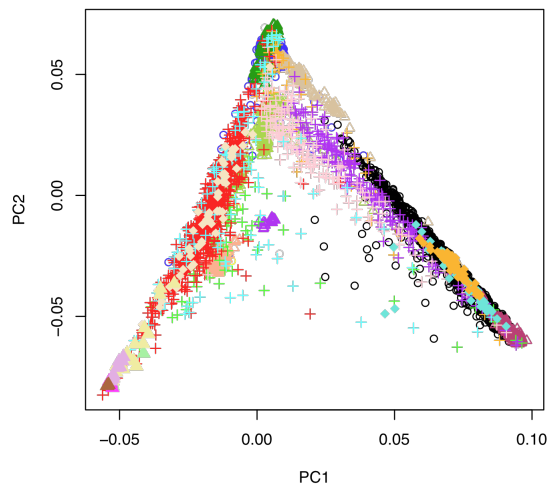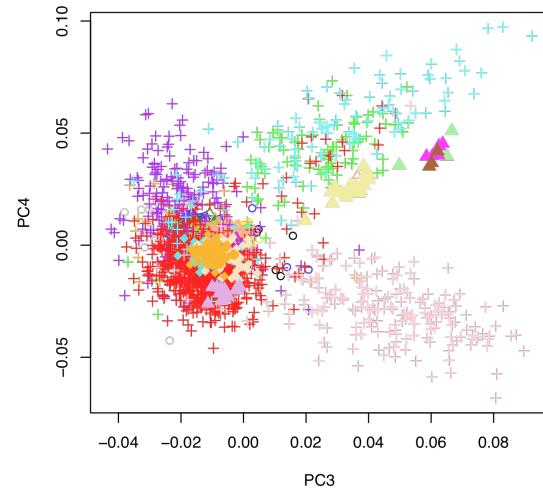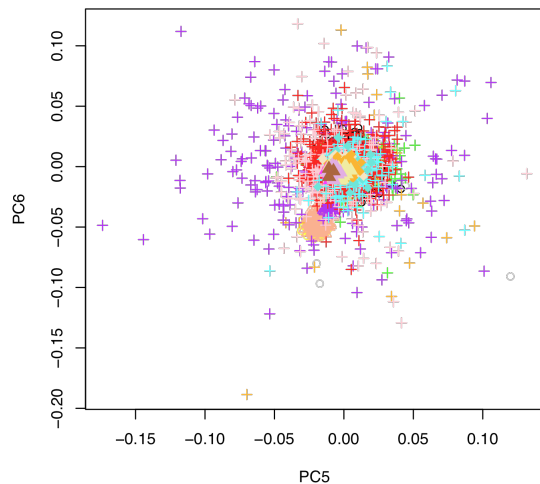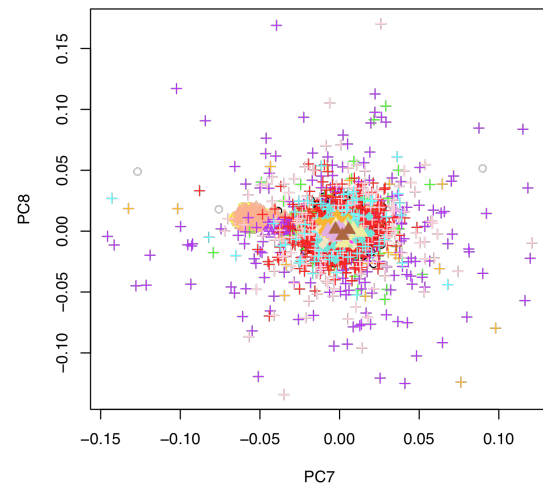

Supplement: Figure S2 — Top eight principal components of ancestry computed in an unrelated subset of 1,374 MESA Hispanic individuals, with projection to an unrelated subset of individuals from the other MESA ethnic groups, in addition to samples from the HapMap and HGDP. Individuals are labeled according to group inclusion: “MESAHispNOS”="MESA Hispanic, Other or Unspecified country/region or origin”, other labels are self-explanatory. “HapHGDPAfrica” includes LWK and YRI from the HapMap, as well as African samples from the HGDP. “HapHGDPEurope” includes CEU and TSI from the HapMap and European samples from the HGDP. “HapHGDPCSAsia” includes GIH from the HapMap and Central/South Asian samples from the HGDP. “HapHGDPEastAsia” includes CHB, CHD, and JPT from the HapMap and East Asian samples from the HGDP. All other labels are self-explanatory. (PDF) [file pgen.1002640.s002.pdf]

CentralAmer  
Cuba  
Dominican  
Mexico  
PuertoRico  
SouthAmer

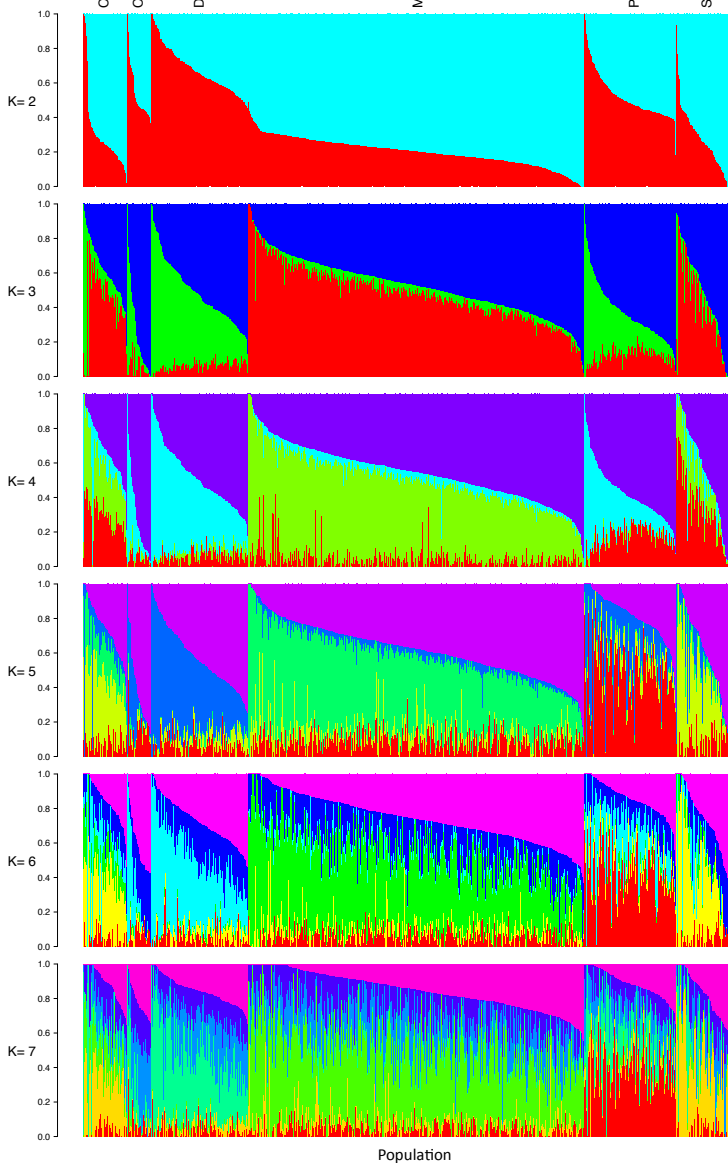

Supplement: Figure S3 — Individual-level proportion of ancestry estimates from model-based clustering analysis in ADMIXTURE for 1,374 unrelated individuals of self-reported Hispanic origin from the Multi-Ethnic Study of Atherosclerosis (MESA), shown for K values 2 through 7. (PDF) [file pgen.1002640.s003.pdf]

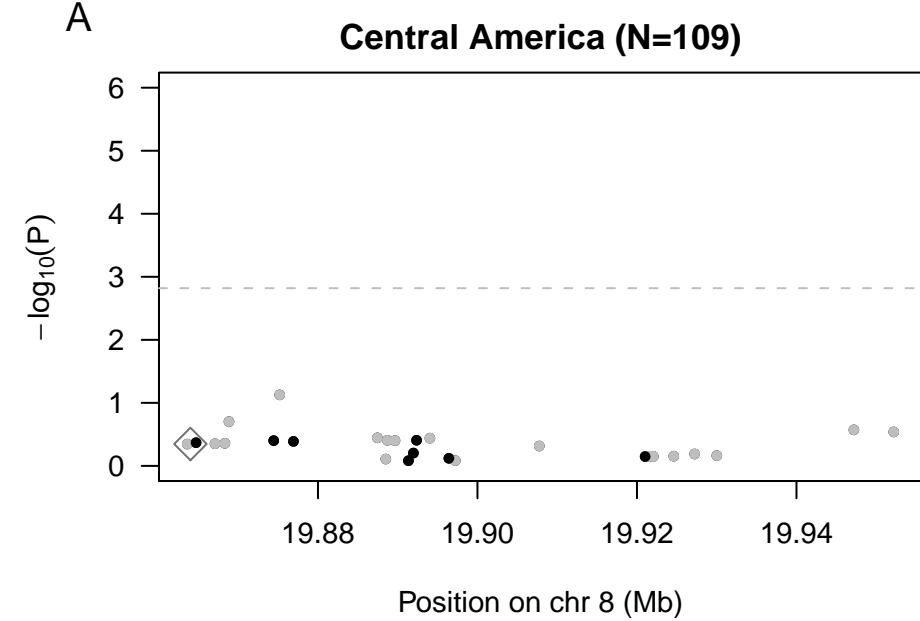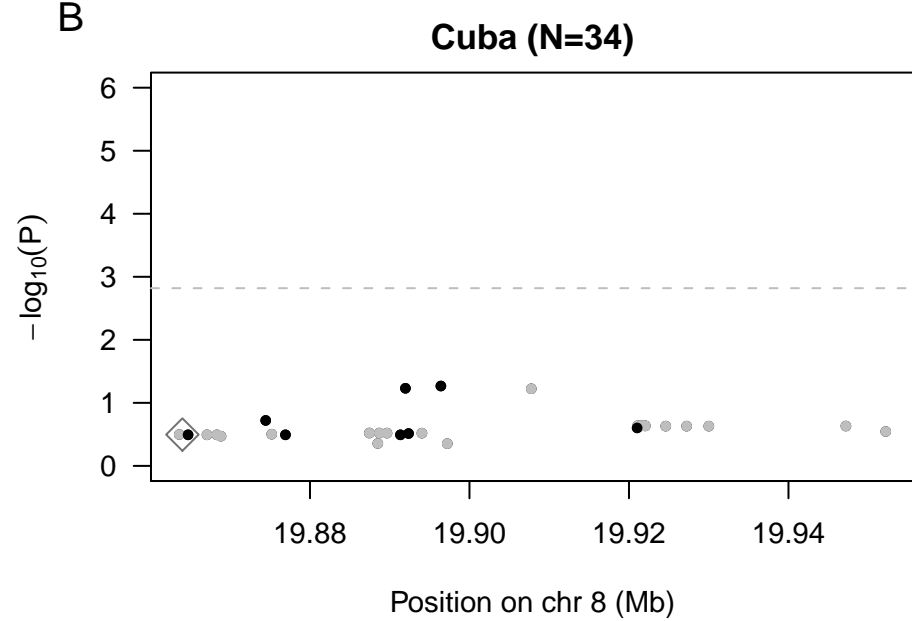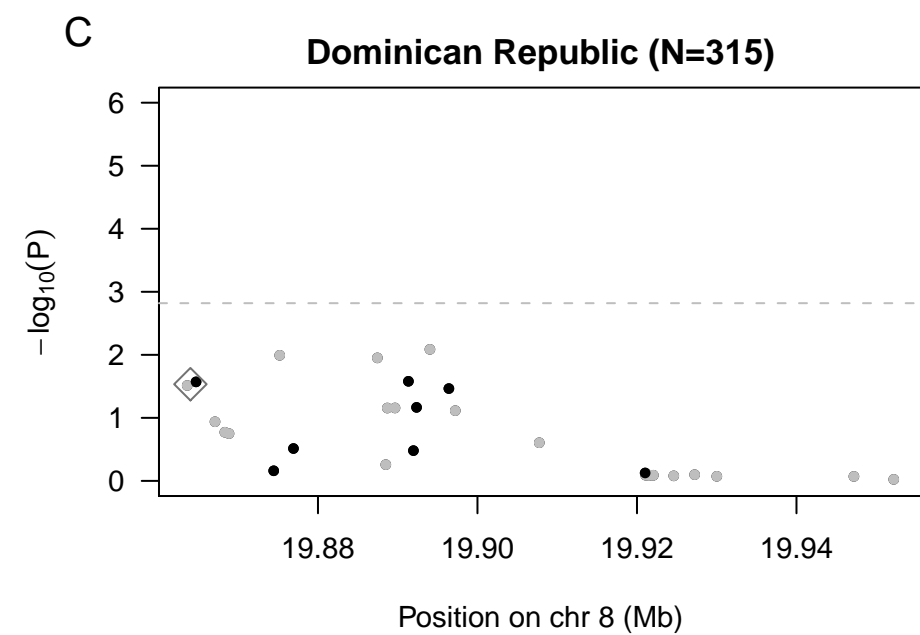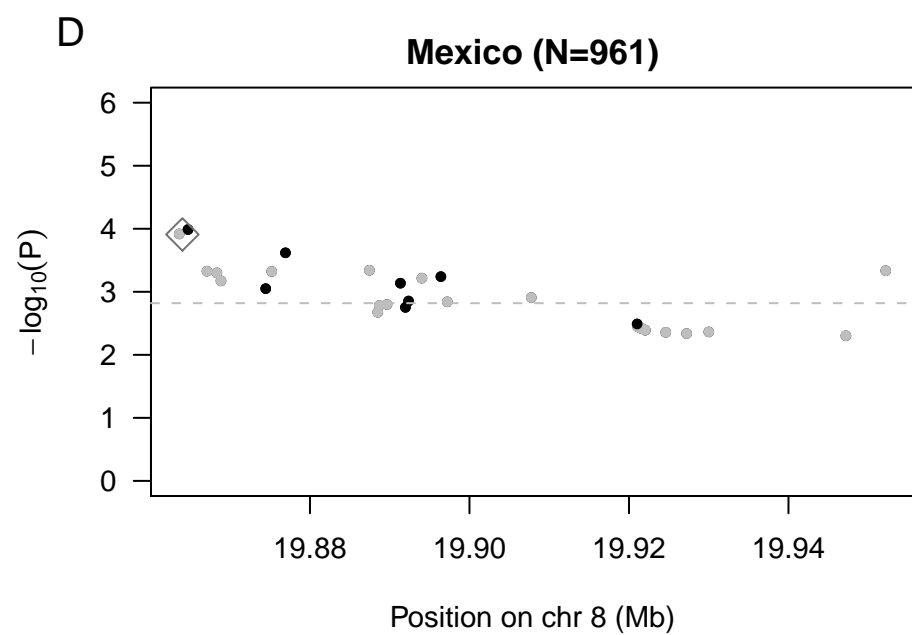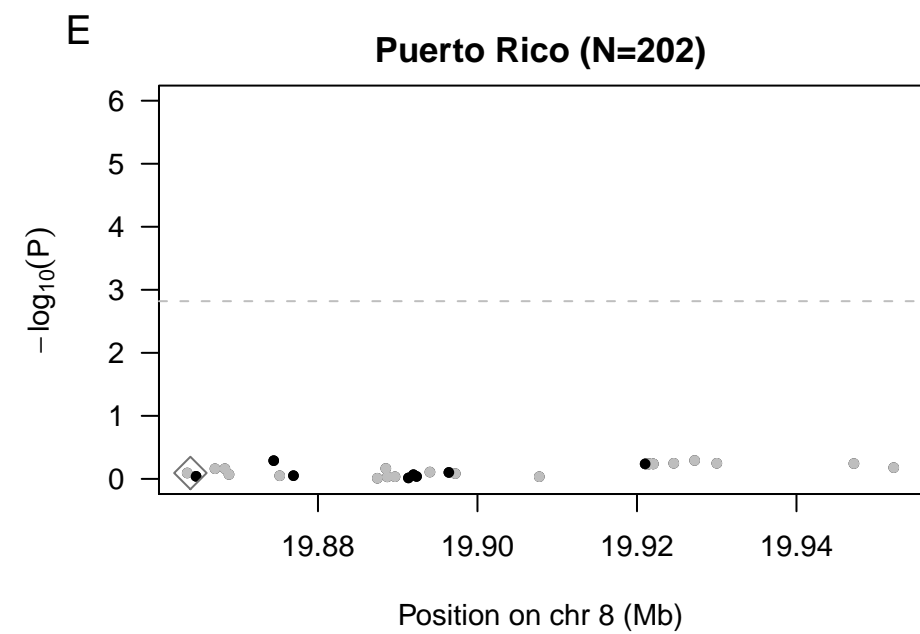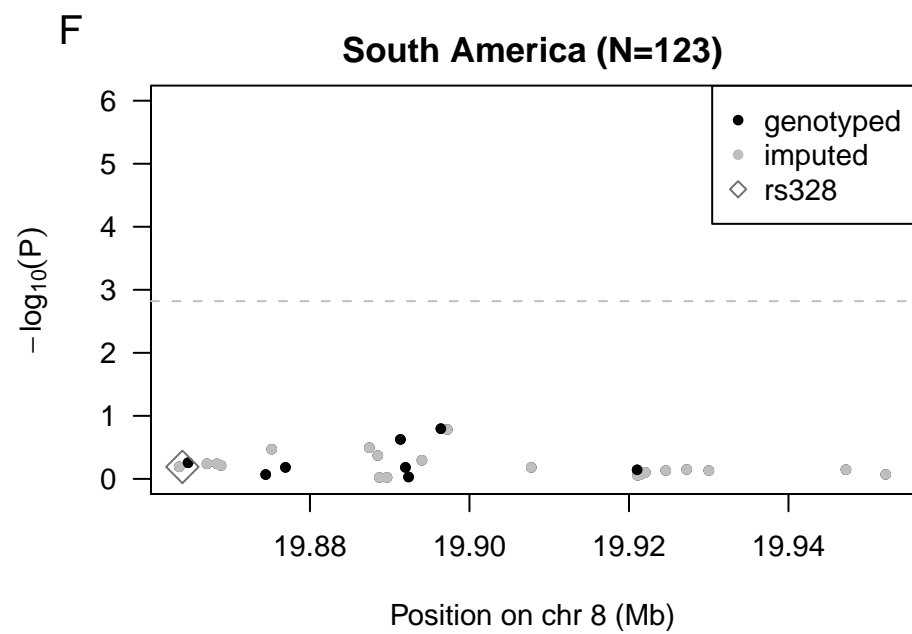

Supplement: Figure S4 — Summary of regional association for SNPs in the LPL gene region with triglycerides (modeled on a log scale). Strength of association versus SNP position on chromosome 8 based on stratified analyses for self-reported country/region of origin corresponding to (A) Central America, (B) Cuba, (C) the Dominican Republic, (D) Mexico, (E) Puerto Rico, and (F) South America. Genotyped SNPs are indicated as solid black dots, imputed SNPs as solid gray dots, the imputed SNP rs328 as an open gray diamond, and horizontal dashed gray lines indicate a conservative Bonferroni-threshold for statistical significance based on multiple testing of 33 SNPs. (PDF) [file pgen.1002640.s004.pdf]

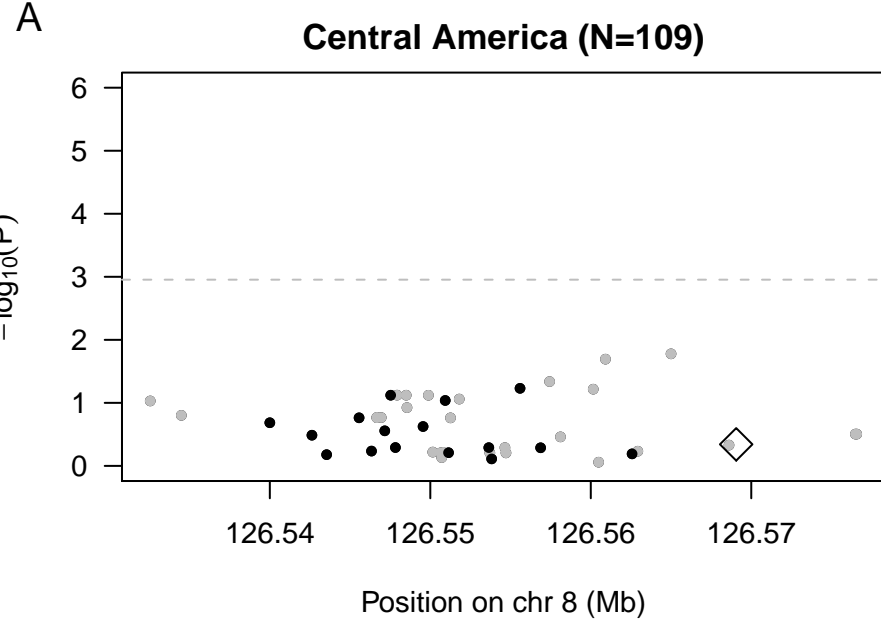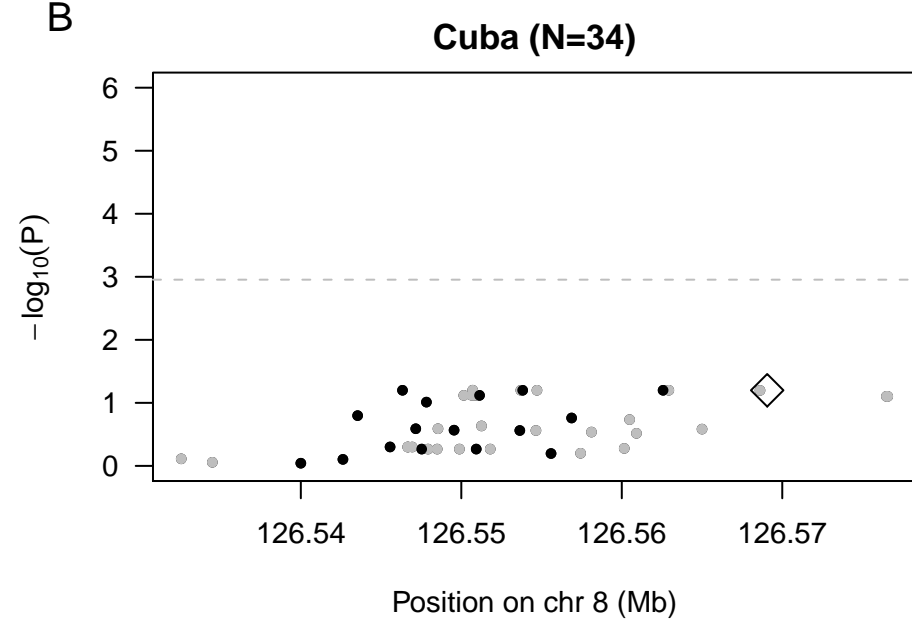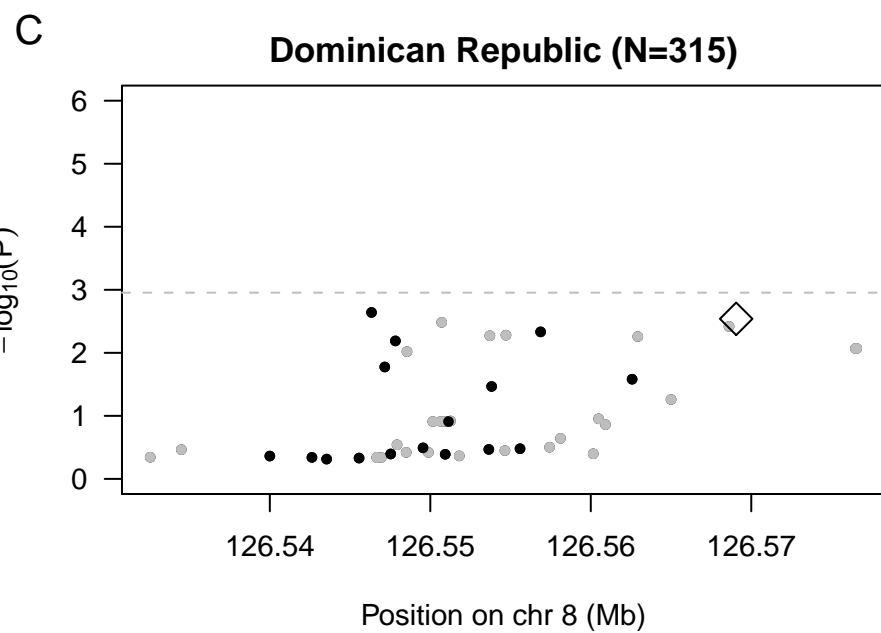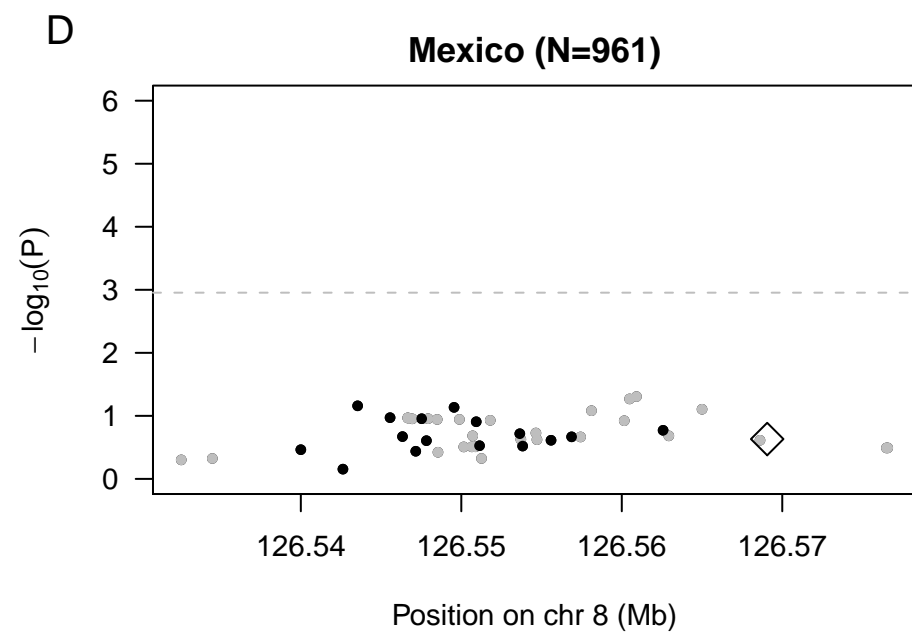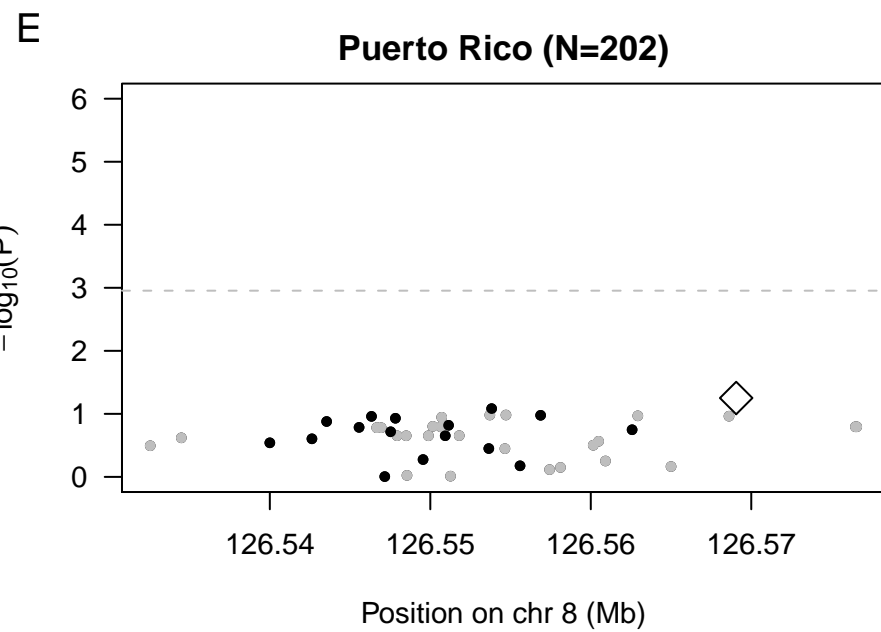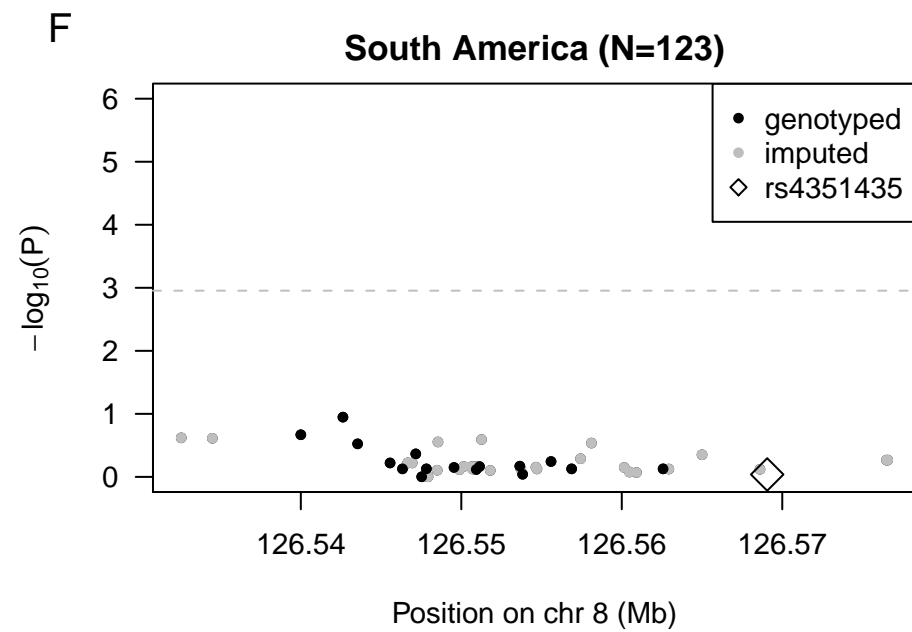

Supplement: Figure S5 — Summary of regional association for SNPs in the TRIB1 gene region with triglycerides (modeled on a log scale). Strength of association versus SNP position on chromosome 8 based on stratified analyses for self-reported country/region of origin corresponding to (A) Central America, (B) Cuba, (C) the Dominican Republic, (D) Mexico, (E) Puerto Rico, and (F) South America. Genotyped SNPs are indicated as solid black dots, imputed SNPs as solid gray dots, the genotyped SNP rs4351435 as an open black diamond, and horizontal dashed gray lines indicate a conservative Bonferroni-threshold for statistical significance based on multiple testing of 45 SNPs. (PDF) [file pgen.1002640.s005.pdf]

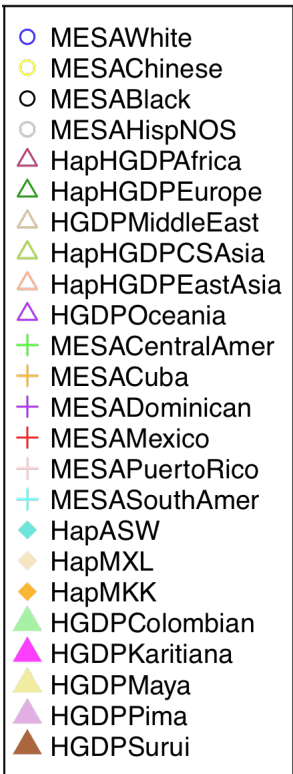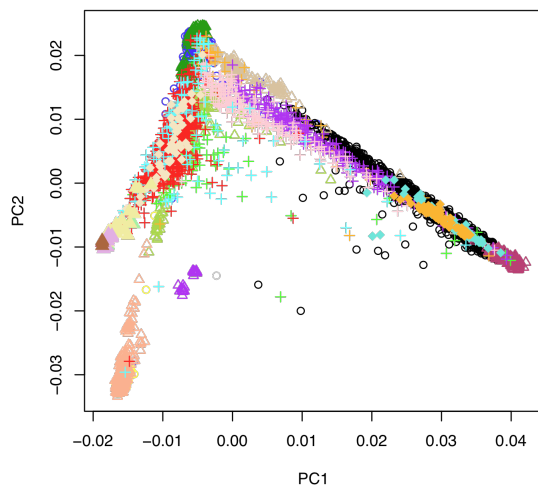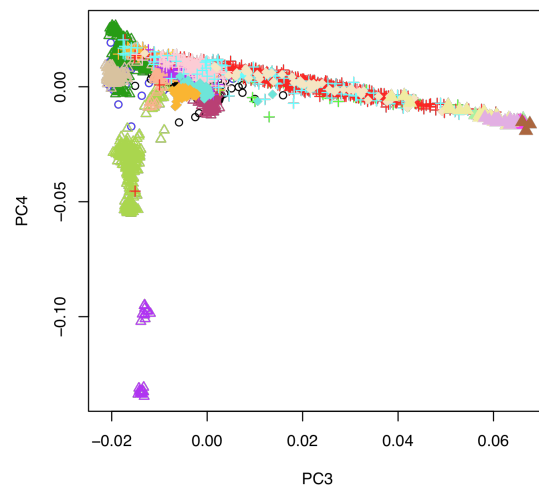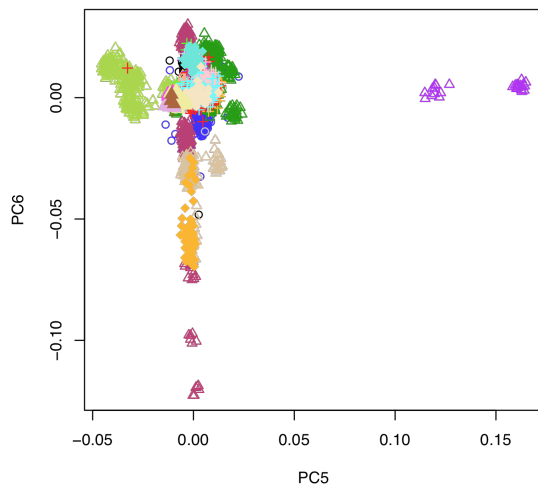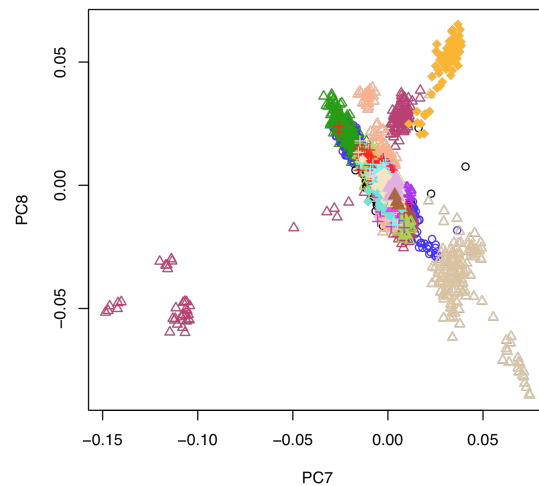

Supplement: Figure S6 — Top eight principal components of ancestry computed in pooled analysis of unrelated MESA Hispanic individuals together with HapMap and HGDP samples, with projection to an unrelated subset of individuals from the other MESA ethnic groups. Individuals are labeled according to group inclusion: “MESAHispNOS”="MESA Hispanic, Other or Unspecified country/region or origin”, other labels are self-explanatory. “HapHGDPAfrica” includes LWK and YRI from the HapMap, as well as African samples from the HGDP. “HapHGDPEurope” includes CEU and TSI from the HapMap and European samples from the HGDP. “HapHGDPCSAsia” includes GIH from the HapMap and Central/South Asian samples from the HGDP. “HapHGDPEastAsia” includes CHB, CHD, and JPT from the HapMap and East Asian samples from the HGDP. All other labels are self-explanatory. (PDF) [file pgen.1002640.s006.pdf]

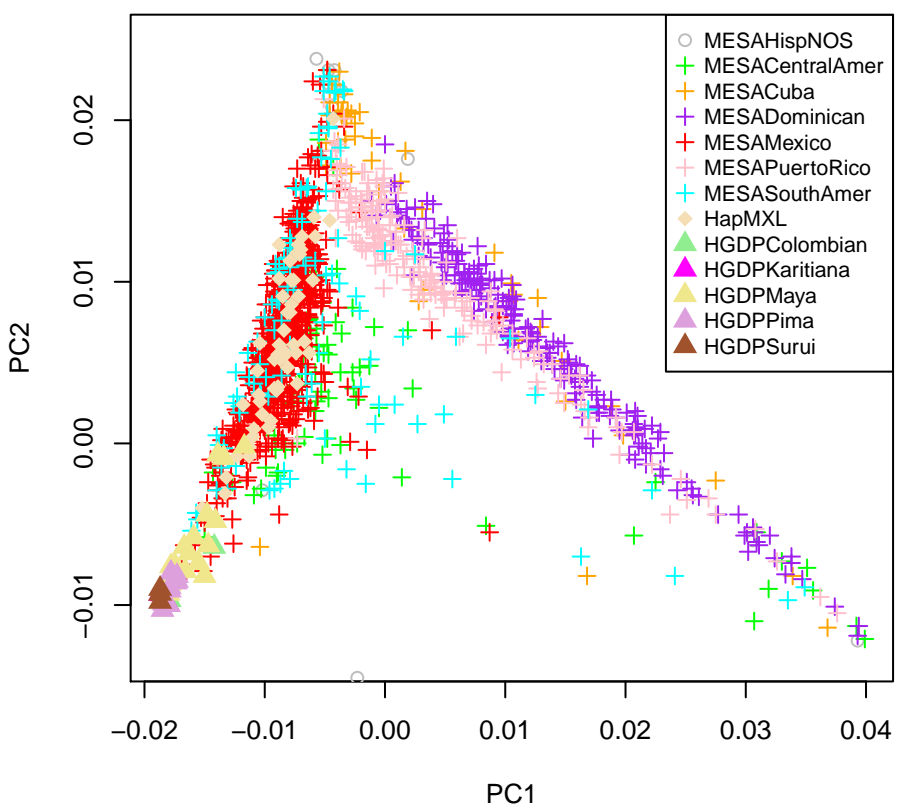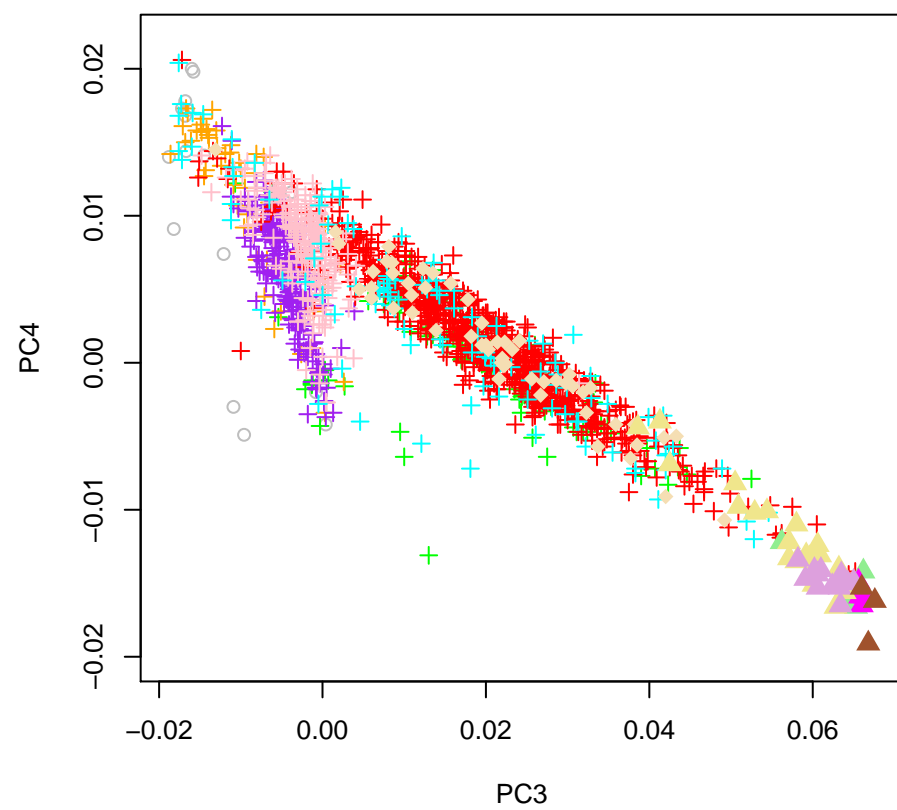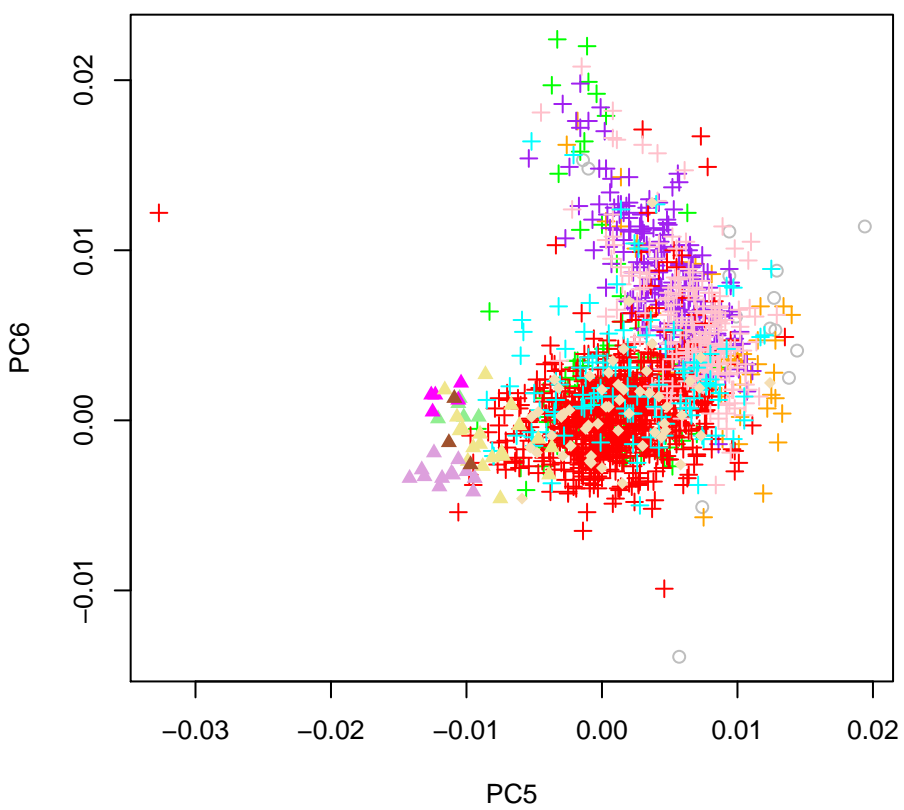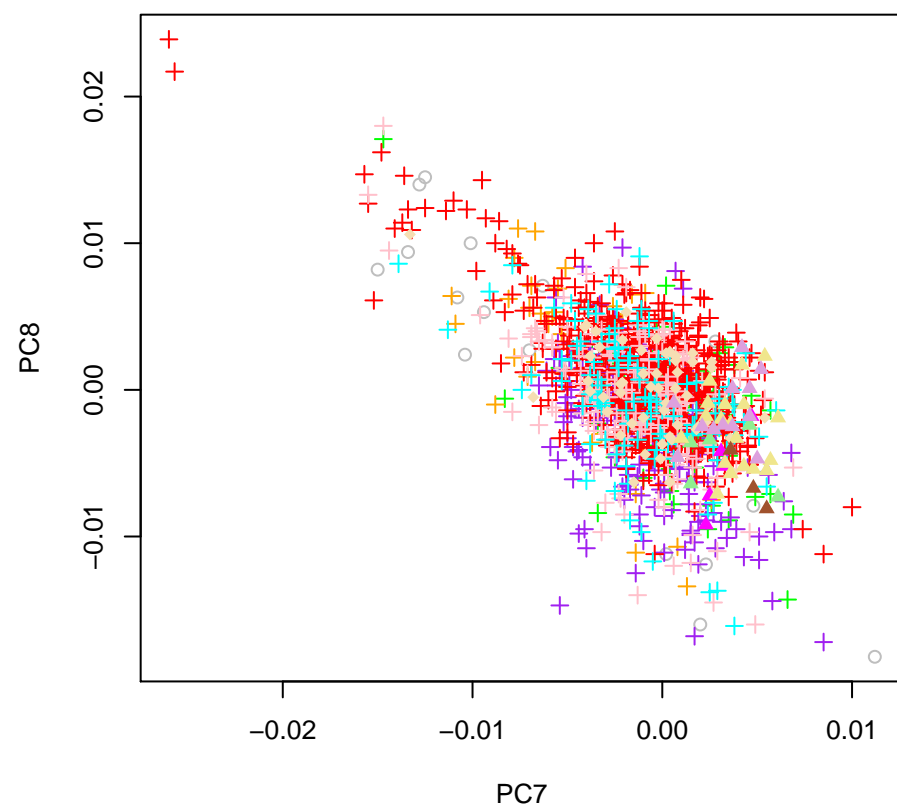

Supplement: Figure S7 — Top eight principal components of ancestry computed in pooled analysis of unrelated MESA Hispanic individuals together with HapMap and HGDP samples. Results are displayed for an unrelated subset of individuals from the MESA Hispanic cohort and key reference populations from the HapMap and HGDP. (PDF) [file pgen.1002640.s007.pdf]
